# Tbx20 Induction Promotes Zebrafish Heart Regeneration by Inducing Cardiomyocyte Dedifferentiation and Endocardial Expansion

**DOI:** 10.3389/fcell.2020.00738

**Published:** 2020-08-04

**Authors:** Yabo Fang, Kaa Seng Lai, Peilu She, Jianjian Sun, Wufan Tao, Tao P. Zhong

**Affiliations:** ^1^State Key Laboratory of Genetic Engineering, School of Life Sciences, Fudan University, Shanghai, China; ^2^Shanghai Key Laboratory of Regulatory Biology, Institute of Molecular Medicine, School of Life Sciences, East China Normal University, Shanghai, China

**Keywords:** Tbx20, heart regeneration, cardiomyocyte dedifferentiation, endocardium, BMP signaling, zebrafish

## Abstract

Heart regeneration requires replenishment of lost cardiomyocytes (CMs) and cells of the endocardial lining. However, the signaling regulation and transcriptional control of myocardial dedifferentiation and endocardial activation are incompletely understood during cardiac regeneration. Here, we report that T-Box Transcription Factor 20 (Tbx20) is induced rapidly in the myocardial wound edge in response to various sources of cardiac damages in zebrafish. Inducing Tbx20 specifically in the adult myocardium promotes injury-induced CM proliferation through CM dedifferentiation, leading to loss of CM cellular contacts and re-expression of cardiac embryonic or fetal gene programs. Unexpectedly, we identify that myocardial Tbx20 induction activates the endocardium at the injury site with enhanced endocardial cell extension and proliferation, where it induces the endocardial Bone morphogenetic protein 6 (Bmp6) signaling. Pharmacologically inactivating endocardial Bmp6 signaling reduces expression of its targets, Id1 and Id2b, attenuating the increased endocardial regeneration in *tbx20*-overexpressing hearts. Altogether, our study demonstrates that Tbx20 induction promotes adult heart regeneration by inducing cardiomyocyte dedifferentiation as well as non-cell-autonomously enhancing endocardial cell regeneration.

## Introduction

Adult mammalian hearts have limited regeneration capacity in response to cardiac damage. Injured hearts lose cardiac muscle and replace with fibrotic scar tissue, ultimately leading to arrhythmia and heart dysfunction (Xin et al., 2013; Tzahor and Poss, 2017). However, zebrafish and neonatal murine hearts exhibit increased regeneration capacity after various insults (Kikuchi and Poss, 2012; Li et al., 2015). Heart regeneration occurs through diverse mechanisms including activation of epicardial, myocardial, or endocardial tissues (Kikuchi and Poss, 2012). Although various mitogenic factors and signaling pathways have been identified to enhance heart regeneration (Kim et al., 2010; Heallen et al., 2013; D’Uva et al., 2015; Gemberling et al., 2015; Wang et al., 2015; Liu and Zhong, 2017; Mohamed et al., 2018; Singh et al., 2018), the signaling and transcriptional control of heart regeneration by myocardial dedifferentiation and endocardial activation are largely unknown. Understanding injury-induced heart regeneration will provide therapeutic strategies to empower regenerative capacity to the diseased human heart.

T-box transcription factor 20 (Tbx20), a key cardiac transcriptional factor, is required for heart development and homeostasis (Greulich et al., 2011). In humans, the heterozygous nonsense or missense mutations of *TBX20* are associated with diverse cardiac pathologies such as dilated cardiomyopathy, atrial septal defect, cardiac valve defects and tetralogy of Fallot (Kirk et al., 2007; Huang et al., 2017). Both *tbx20*-null zebrafish and mice are embryonic lethal and exhibit deleterious cardiovascular malformations with defects of CM proliferation and heart tube looping (Cai et al., 2005; Singh et al., 2005; Lu et al., 2017). *Tbx20*-deficient mice also display defects in cardiac chamber differentiation, endocardial cushion formation, and atrioventricular canal (AVC) patterning (Singh et al., 2005; Stennard et al., 2005; Shelton and Yutzey, 2007; Cai et al., 2013). Myocardial-specific *Tbx20* ablation in adult mice leads to thinner ventricle wall and cardiomyopathy accompanied with arrhythmias (Shen et al., 2011; Sakabe et al., 2012). Conversely, inducible *tbx20* overexpression in embryonic cardiomyocytes leads to increased CM proliferation and thickening of the myocardium in adult hearts (Chakraborty et al., 2013). Myocardial-specific *tbx20* overexpression in zebrafish embryos also results in enlarged heart with both increased cardiac progenitor cell formation and the proliferation of differentiated CMs (Lu et al., 2017). Recent studies report that *Tbx20* overexpression in adult mouse hearts after myocardial infarction increases CM proliferation in the injury border zone and improves cardiac function recovery (Xiang et al., 2016). Despite that previous studies demonstrate essential roles of Tbx20 transcription factor during heart development, injury repair and congenital heart disease, it is currently not understood whether and how endocardial cells respond to Tbx20 induction in the myocardium after cardiac damage, and the extent to which Tbx20 regulates CM dedifferentiation and proliferation during heart regeneration.

The heart develops through generation of CMs and tightly associated endocardial cells (Staudt and Stainier, 2012). Endocardial cells represent a subset of a larger endothelial cell pool (Harris and Black, 2010). During development, the endocardium is organized into arterial and venous subpopulations with comparable gene expression profiles (Staudt and Stainier, 2012). Endocardial differentiation and growth occur without an accretion of external cells, in a manner independent of vascular endothelial growth factor (VEGF) signaling (Dietrich et al., 2014). After cardiac injury, activated endocardium coincides with changes in cell morphology and gene expression (Kikuchi et al., 2011b). The activation and maturation of the endocardium require Notch signaling, which supports myocardial regeneration (Munch et al., 2017; Zhao et al., 2019). However, no signaling factor or molecular program has been shown to be essential for endocardial cell proliferation during zebrafish heart regeneration.

In this report, we have determined Bmp6 signaling as an early endocardial injury-response to myocardial Tbx20 induction, which promotes endocardial cell regeneration, a previously unrecognized mechanism. Tbx20 is also sufficient to induce injury-induced CM dedifferentiation, thus stimulate CM proliferation. Overall, our findings reveal novel roles and distinct mechanisms of myocardial Tbx20-mediated network in governing cardiac muscle production and endocardial cell proliferation during heart regeneration.

## Results

### Tbx20 Is Induced in the Regenerating Zebrafish Heart Following Injury

**FIGURE 1:**
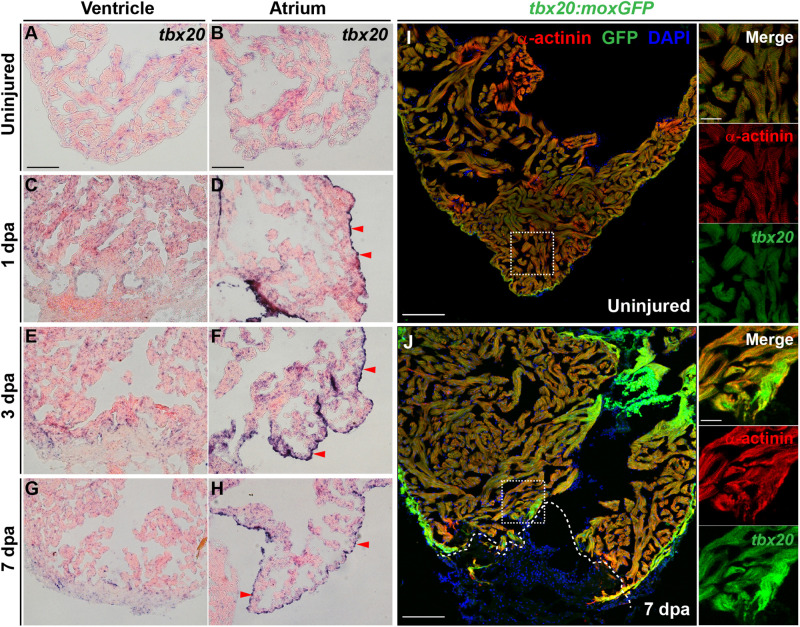
Cardiac injury triggers a localized increase in *tbx20* expression. **(A–H)** Representative images of ISH for *tbx20* on the heart sections of uninjured **(A,B)** and injured ventricles and atriums at different time points as indicated **(C–H)**. Red arrowheads indicate atrial epicardium. **(I,J)** Confocal images of uninjured **(I)** and 7 dpa **(J)** heart sections from *Tg*(*tbx20:moxGFP*) zebrafish immunostained for GFP and α-actinin (red). DAPI marks nuclei. Boxed areas are magnified on the right panels. Dashed lines in **(J)** demarcate amputation planes. Scale bar: 100 μm **(A–J)**; 20 μm (inset images).

To define the spatiotemporal expression pattern of T-Box Transcription Factor 20 (Tbx20) during adult heart regeneration, we first evaluated *tbx20* expression during the window of zebrafish cardiac regeneration by *in situ* hybridization (ISH). In the uninjured adult heart, we detected faint *tbx20* expression in the ventricles and atriums (Figures 1A,B). Within 1 day post amputation (dpa), *tbx20* expression was induced in both the myocardium and atrial epicardium (Figures 1C,D). By 3 dpa, *tbx20* was strongly upregulated in the ventricular and the atrial myocardium (Figures 1E,F). Specifically, more *tbx20*^+^ cells were accumulated in the injury border zone of the ventricle compared to the remote (uninjured) zone at 3 dpa and 7 dpa (Figures 1E–H). Furthermore, qPCR analyses validated the marked upregulation of *tbx20* at the border zone of injured ventricles and the atrium at 1 dpa, 3 dpa and 7 dpa (Supplementary Figure S1A). The induction of *tbx20* at the injury border zone and the atrial epicardium was also detectable at 5 days post cardiac cryoinjury (dpci), an independent injury approach (Supplementary Figures S1B–E). However, *tbx20* induction at the atrial epicardium was hardly detectable at uninjured hearts (Figure 1B and Supplementary Figure S1D). As controls, *tbx20* transcripts were not detectable in uninjured and injured hearts from 1 dpa to 7 dpa using *tbx20* sense probes (Supplementary Figures S1F–M), confirming the specificity of *tbx20* upregulation in the ventricle and the atrial epicardium after cardiac damage.

To unambiguously define the myocardial expression of *tbx20*, we generated a *Tg(tbx20: moxGFP)* transgenic zebrafish expressing monomeric oxidizing GFP (moxGFP) driven by a *tbx20* promoter-based upstream region. We were able to detect CMs that expressed weak moxGFP in the ventricle of uninjured hearts after co-immunostaining for GFP and α-actinin (Z-disk marker) (Figure 1I and Supplementary Figure S1N). In the injured adult heart, extensive and stronger moxGFP signals were observed in the ventricles, as well as at the injury border zone by 7 dpa and 5 dpci, respectively, (Figure 1J and Supplementary Figure S1O). While moxGFP signals were hardly detectable in the epicardium in uninjured hearts (Supplementary Figures S1P, S2A), strong GFP signals colocalized with epicardial marker pan-cytokeratin (PCK) were detectable in the atrial epicardium of injured hearts (Supplementary Figures S1Q, S2B). These results indicate the *tbx20* expression is induced in the ventricular myocardium and the atrial epicardium after cardiac injury, consistent with ISH analyses.

### Inducible *tbx20* Overexpression in the Adult Myocardium Promotes CM Proliferation and Heart Regeneration

**FIGURE 2:**
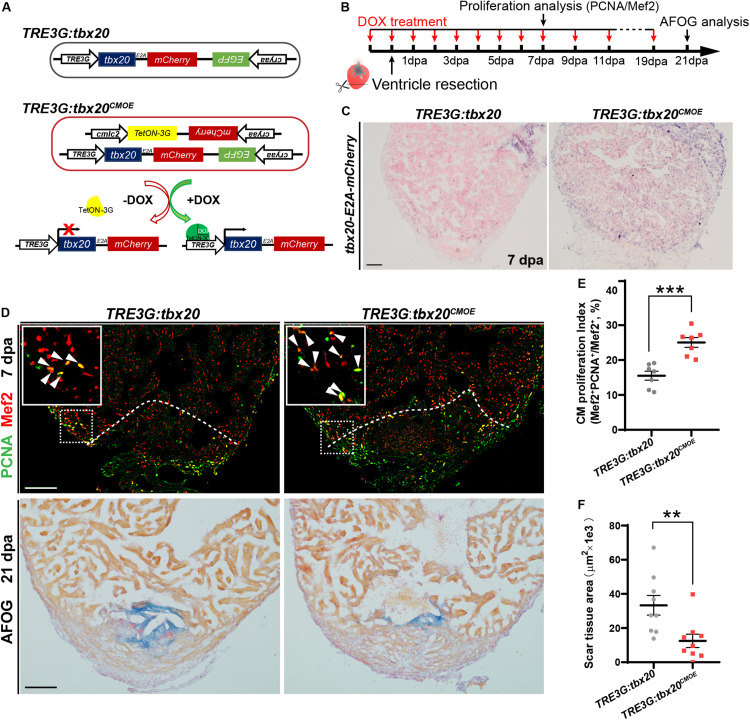
Myocardial *tbx20* overexpression in adult hearts promotes CM proliferation and reduces fibrotic scars. **(A)** Schematic diagram of transgenes of the control line *Tg*(*TRE3G:tbx20*) and the inducible myocardial *tbx20* overexpression line *Tg*(*TRE3G:tbx20^*CMOE*^*) with DOX treatment. **(B)** Experimental strategy employed to induce myocardial *tbx20* overexpression during heart regeneration. **(C)** Representative images of ISH with *tbx20-E2A-mCherry* probe on 7 dpa heart sections from DOX treated *Tg*(*TRE3G:tbx20*) and *Tg*(*TRE3G:tbx20^*CMOE*^*) fish. Scale bar: 100 μm. **(D)** Upper panels: Representative confocal fluorescence images of heart sections from 7 dpa zebrafish immunostained for PCNA (green) and Mef2 (red). Insets showing high-magnification images of proliferating cardiomyocytes, arrowheads indicate PCNA^+^Mef2^+^ cells. Lower panels: Representative images of heart sections from 21 dpa fish stained with AFOG, muscle stained brown, collagen is blue and fibrin is red. The genotypes of fish were indicated above the images. Scale bar: 100 μm. **(E)** Quantification of CM proliferation at border zone and injury site on 7 dpa heart sections (*n* = 7 in each group). **(F)** Quantification of scar area at 21 dpa (*n* = 9 in each group). The fish in **(C–F)** were treated with DOX as indicated in **(B)**. Each value in **(E,F)** represents mean ± SEM, **p < 0.01, ***p < 0.001.

To investigate the biological functions of *tbx20* during heart regeneration, we generated transgenic zebrafish, *Tg(cmlc2:TetON-3G; cryaa:mCherry)* carrying a CM-specific *TetON-3G* and a lens-specific marker *mCherry*, as well as *Tg(TRE3G:tbx20-E2A-mCherry; cryaa:EGFP)* containing a doxycycline (DOX)-inducible *tbx20-E2A-mCherry* and a lens-specific marker *EGFP*, hereafter referred to as *TRE3G:tbx20* (Figure 2A). By crossing this two lines, we established a double transgenic zebrafish strain, *Tg*(*cmlc2*:*TetON-3G*, *cryaa:mCherry*; *TER3G:tbx20*-*E2A-mCherry, cryaa:EGFP*), referred to as *TRE3G:tbx20^*CMOE*^*, permitting conditional expression of *tbx20* specifically in CMs induced by DOX (Figure 2A). After daily administration of adult zebrafish with DOX (50 mg/L) from the day before cardiac resection to the day of examination (Figure 2B), *TRE3G:tbx20^*CMOE*^* fish displayed mCherry fluorescence only in myocardium and no ectopic mCherry expression elsewhere (Supplementary Figures S3A–F). Lack of leaking expression of *tbx20-E2A-mCherry* in *TRE3G:tbx20^*CMOE*^* fish was further validated by our ISH analyses showing strong *tbx20* induction in myocardium in DOX-treated *TRE3G:tbx20^*CMOE*^* fish (Figure 2C and Supplementary Figure S3I), but only weak signal in myocardium in *TRE3G:tbx20^*CMOE*^* fish without DOX treatment, which is comparable to that in DOX-treated *TRE3G:tbx20* fish (Supplementary Figures S3G,H).

During zebrafish heart regeneration, newly formed CMs primarily come from the proliferation of pre-existing CMs (Jopling et al., 2010; Kikuchi and Poss, 2012). To assess whether *tbx20* promotes CM proliferation during heart regeneration, *TRE3G:tbx20^*CMOE*^* and control *TRE3G*:*tbx20* zebrafish were treated with DOX (Figure 2B), and subjected to ventricular apex resection next day. Heart sections at 7 dpa, a time point when CM proliferation peaks, (Wills et al., 2008; Wang et al., 2011), were immunostained with antibodies against proliferation marker PCNA and the CM nuclear marker Mef2. The results revealed that CM proliferation in injured *TRE3G:tbx20^*CMOE*^* hearts markedly increased by ∼61%, compared with that in control hearts (25.1 ± 1.4% versus 15.5 ± 1.3%) (Figures 2D,E). By contrast, *tbx20* overexpression had no discernible effects on CM proliferation in uninjured adult hearts after 7 days of DOX treatment in which PCNA^+^Mef2^+^ CMs were not detectable in *tbx20* overexpressing hearts and control hearts (Supplementary Figure S4). Collectively, these results demonstrate that myocardial overexpressing *tbx20* stimulates injury-induced CM proliferation.

Given that increased *tbx20* expression is able to enhance CM proliferation at injured sites at an early regeneration stage, we reasoned that long-term high transcription level of *tbx20* reduced fibrotic scars and hastened wound healing. To test this possibility, heart regeneration of DOX-treated *TRE3G:tbx20^*CMOE*^* and *TRE3G:tbx20* fish at 21 dpa were evaluated using Acid Fuchsin-Orange G (AFOG) staining of heart cryosections. Our study showed that hearts from *TRE3G:tbx20^*CMOE*^* fish were evidenced by contiguous cardiac muscle formation and reduced fibrotic scars at the injured ventricle apex, whereas hearts from *TRE3G:tbx20* fish remained variable of prominent scar tissues (Figures 2D,F).

### Myocardial-Specific *tbx20* Overexpression Enhances Injury-Induced CM Dedifferentiation

**FIGURE 3:**
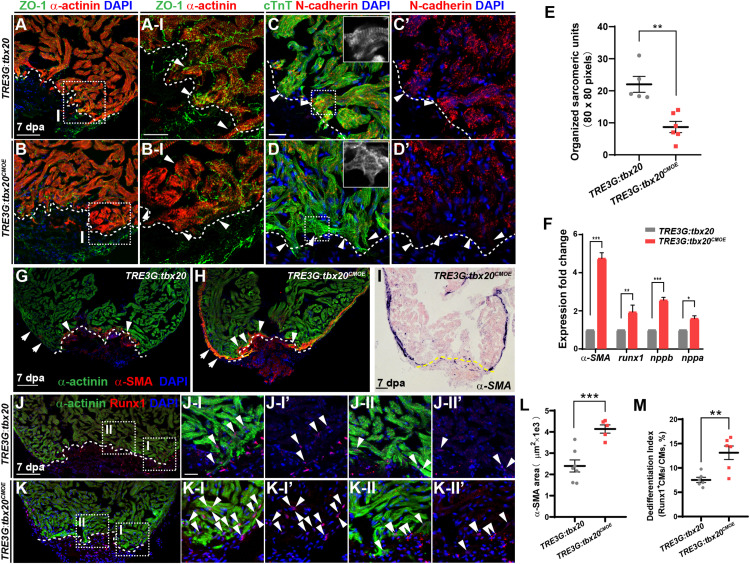
*tbx20* overexpression in the adult myocardium enhances CM dedifferentiation after injury. **(A–D,G,H,J,K)** Representative confocal fluorescence images of sections of injured ventricles from *Tg*(*TRE3G:tbx20*) and *Tg*(*TRE3G:tbx20^*CMOE*^*) zebrafish co-stained with antibodies against ZO-1 (green) and α-actinin (red) **(A,B)**, cTnT (green) and N-cadherin (red) **(C,D)**, α-SMA (red) and α-actinin (green) **(G,H)**, and Runx1 (red) and α-actinin (green) **(J,K)**. DAPI was used to stain nuclei. Boxed areas in **(A,B,J,K)** are magnified on the right with split channels. Insets in **(C,D)** show enlarged images of the dashed boxes. Arrowheads in **(A-I, B-I)** indicate CMs with disassembled sarcomeric structure in the border zone adjacent to the injury site. Arrowheads in **(C,D)** point to CMs adjacent to the injury site. Arrows and arrowheads in **(G,H)** indicate α-SMA^+^ cells in the regenerating compact layer and trabecular layer, respectively. Arrowheads in **(J,K)** indicate Runx1^+^α-actinin^+^ CMs. **(E)** Quantification of organized sarcomeric units in cTnT-labeled myocardium (80 × 80 pixels) in border zone from 7 dpa ventricle sections of *Tg*(*TRE3G:tbx20*) **(C**, *n* = 5) and *Tg*(*TRE3G:tbx20^*CMOE*^*) (**D**, *n* = 6) zebrafish. **(F)** Statistical analyses of qPCR for *α-SMA*, *runx1*, *nppb* and *nppa* in the injured ventricle apices from *Tg*(*TRE3G:tbx20*) and *Tg*(*TRE3G:tbx20^*CMOE*^*) zebrafish at 7 dpa. **(I)** Representative images of ISH with *α-SMA* on 7 dpa heart sections from *Tg*(*TRE3G:tbx20^*CMOE*^*) fish. **(L)** Dot plot showing the area of α-SMA stained on heart sections in **(G**, *n* = 7) and **(H**, *n* = 5). **(M)** Dot plot showing the percentage of Runx1^+^α-actinin^+^ cells in the border zone in **(J,K)**, *n* = 6 in each group. All fish were treated with DOX as described in Figure 2B and hearts were harvested at 7 dpa. Dashed lines delineate injured area. The values in **(E,F,L,M)** are mean ± SEM, ^∗^*p* < 0.05, ^∗∗^*p* < 0.01, ^∗∗∗^*p* < 0.001. Scale bar: 50 μm **(A,B)**; 20 μm **(A-I,B-I,C-D’,J-I-K-II”)**; 100 μm **(G–K)**.

Cardiomyocyte dedifferentiation, a transition from mature state to immature state, is a mechanism to ensure subsequent CM proliferation that naturally occurs in response to cardiac injury in neonatal mouse and adult zebrafish (Jopling et al., 2010; Porrello et al., 2011; D’Uva et al., 2015). This process is characterized by disassembly of sarcomeric structure, loss of cell-cell adhesion and re-expression of cardiac embryonic, fetal or progenitor genes (Kubin et al., 2011; D’Uva et al., 2015). Since increased *tbx20* expression enhances CM proliferation and cardiac regeneration (Figure 2), we asked whether *tbx20* cardiac overexpression was capable of boosting CM dedifferentiation following cardiac injury. To test this idea, we determined morphological and molecular changes of CMs in DOX-treated control and *TRE3G:tbx20^*CMOE*^* hearts. We observed a marked reduction of a cell tight junction marker ZO-1 in α-actinin-marked CMs in the injury border zone in *TRE3G:tbx20^*CMOE*^* hearts compared to that in control hearts (Figures 3A–B-I). N-cadherin is a marker of cell-cell adhesion junction localized in the intercalated disks between neighboring CMs (Luo and Radice, 2003; Vite and Radice, 2014; Li et al., 2019). In injured hearts overexpressing *tbx20* in CMs, we observed a reduction of N-cadherin in the border zone of the injured myocardium, indicating a loss of cell-cell contact between CMs (Figures 3C’,D’ and Supplementary Figure S5). Notably, we observed CMs marked by cardiac troponin T (cTnT) in the wound edge exhibited greater extent of sarcomere disassembly that were devoid of myofibril striations in *tbx20*-overexpressing hearts than that in control hearts (Figures 3C–E).

Concomitantly, q-PCR analyses showed that expression of CM dedifferentiation markers, including cardiac fetal markers, *alpha-smooth muscle actin* (*α-SMA*), *natriuretic peptide a* (*nppa*) and *natriuretic peptide b* (*nppb*) (Dirkx et al., 2013; Man et al., 2018), as well as a progenitor cell marker *runx1* (Poling et al., 2012; D’Uva et al., 2015; Wang et al., 2017) were significantly increased in DOX-treated *TRE3G:tbx20^*CMOE*^* hearts compared with control hearts (Figure 3F). We next assessed expression patterns of *α-SMA* (Figures 3G–I,L and Supplementary Figure S6) and Runx1 in injured hearts (Figures 3J,K,M) immunostained with CM marker α-actinin. We observed that α-SMA was markedly induced in the α-actinin-marked myocardial compact layer (Figure 3H and Supplementary Figures S6A, A-I-A-II”), and some of α-SMA signal was detectable in the trabecular layer adjacent to the injury site (Figure 3H and Supplementary Figures S6A, A-II-A-II”) in DOX-treated *TRE3G:tbx20^*CMOE*^* injured hearts. In contrast, *α-SMA* re-expression was restricted to a small injury region in control hearts (Figure 3G). Furthermore, Runx1 was upregulated in more regenerating CMs in *TRE3G:tbx20^*CMOE*^* injured hearts than that in control wounded hearts (Figures 3J,K,M). Similarly, ISH analyses revealed the increased expression of *α-SMA* and *nppb*, as well as cardiac progenitor markers *gata4, gata5* and *hand2* in injured *tbx20*-overexpressing hearts in comparison to control hearts (Figure 3I and Supplementary Figures S7A–H). qPCR analyses validated the upregulation of *gata4, gata5* and *hand2* in *TRE3G:tbx20^*CMOE*^* hearts following ventricular apex resection (Supplementary Figure S7I). Taken together, our data demonstrated that enhanced cardiac *tbx20* expression favors induction of cardiac fetal and progenitor gene programs, resulting in CM dedifferentiation and proliferation during regeneration.

### Tbx20 Mediates Various Genetic Circuits Regulating Zebrafish Heart Regeneration

**FIGURE 4:**
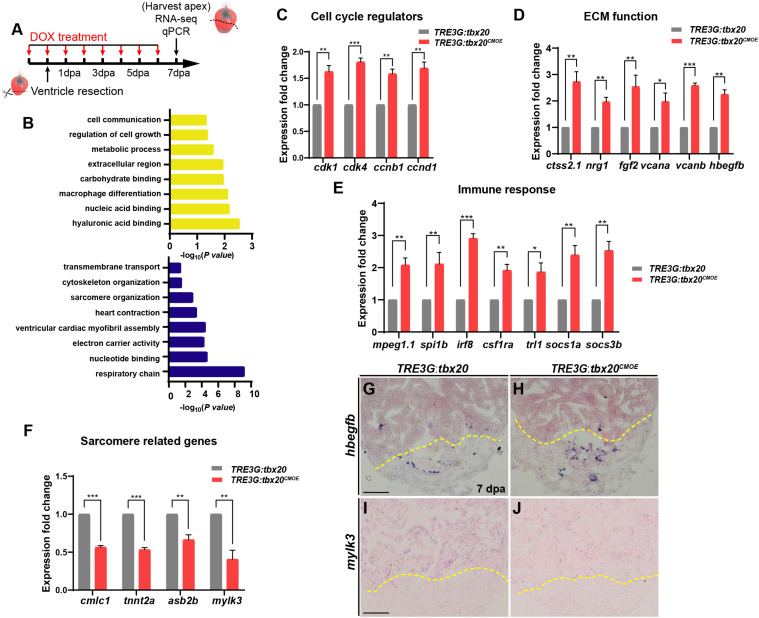
Myocardial *tbx20* overexpression affects expression of various injury responsive genes following heart injury. **(A)** Schematic of DOX treatment, heart injury and tissue collection procedures. RNA samples were extracted from the ventricular apices (below the dashed line). For each group, 6–8 samples were collected, and three independent biological replicates were carried out. **(B)** Significantly enriched gene ontology (GO) analysis based on RNA-seq results revealed upregulated gene categories (upper panel), and downregulated gene categories (lower panel). **(C–F)** Bar graphs indicate qPCR analyses of relative expression levels of genes associated with “Cell cycle regulators” **(C)**, “ECM function” **(D)**, “Immune response” **(E)** and “Sarcomere related genes” **(F)** in ventricular apices from DOX-treated *Tg*(*TRE3G:tbx20^*CMOE*^*) and *Tg*(*TRE3G:tbx20*) at 7 dpa. Data represents three biological replicates. Mean ± SEM, *p < 0.05, **p < 0.01, ***p < 0.001. **(G–J)** Representative images of ISH analyses for *hbegfb* **(G,H)** and *mylk3* **(I,J)** on 7 dpa heart sections from *Tg*(*TRE3G:tbx20^*CMOE*^*) and *Tg*(*TRE3G:tbx20*) zebrafish with DOX treatment. Scale bar: 100 μm.

To decipher the molecular basis in response to cardiac injury with enhanced cardiac *tbx20* expression, we analyzed gene expression profiles of the apical halves of the resected ventricles from DOX-treated *TRE3G:tbx20^*CMOE*^* and *TRE3G:tbx20* zebrafish at 7 dpa (Figure 4A). We found that 1880 genes were differentially expressed in *TRE3G:tbx20^*CMOE*^* hearts (Log FC > 0.5, *p-value* < 0.05) compared to *TRE3G:tbx20* hearts, in which 747 of them were upregulated and 1133 were downregulated (Supplementary Table S1).

GO analyses of the upregulated gene subset revealed enhanced expression of functional categories including “cell growth”, “ECM function”, and “immune response” (Figure 4B and Supplementary Table S2). By qPCR and ISH, we validated the upregulation of cell cycle regulators (*cdk1*, *cdk4*, *ccnb1* and *ccnd1*) (Figure 4C), the extracellular matrix (ECM)-type genes (*ctss2.1*, *nrg1*, *fgf2, vcana, vcanb* and *hbegfb*) (Figures 4D,G,H and Supplementary Table S3) and immune response regulators (*mpeg1.1*, *spi1b*, *irf8*, *csf1ra*, *tlr1*, *socs1a*, *socs3b*) in regenerating hearts overexpressing *tbx20* (Figure 4E and Supplementary Table S3). These results indicate that myocardial induction of *tbx20* participates in heart regeneration not only by upregulating expression of cell cycle regulators, but also modulating genes regulating ECM function and immune response.

GO analyses of downregulated genes revealed an enrichment of factors involved in sarcomere organization, including sarcomere formation genes (*cmlc1*, *desma*, *myom1b*, *tnni1c*, *tnnt2a*) and sarcomere assembling factors (*mylk2*, *mylk3*, *asb2b*) (Supplementary Table S3). We further verified down regulations of *cmlc1*, *tnnt2a*, *asb2b* and *mylk3* in regenerating hearts from DOX-treated *TRE3G:tbx20^*CMOE*^* fish by qPCR and ISH analyses (Figures 4F,I,J). These findings indicate that the regenerating hearts overexpressing *tbx20* caused a reduction of genes regulating sarcomere formation and assembly to favor CM dedifferentiation (Figures 3B,D), consistent with our dedifferentiation experiments (Figure 3).

### Myocardial *tbx20* Overexpression Induces Endocardial Activation and Regeneration

**FIGURE 5:**
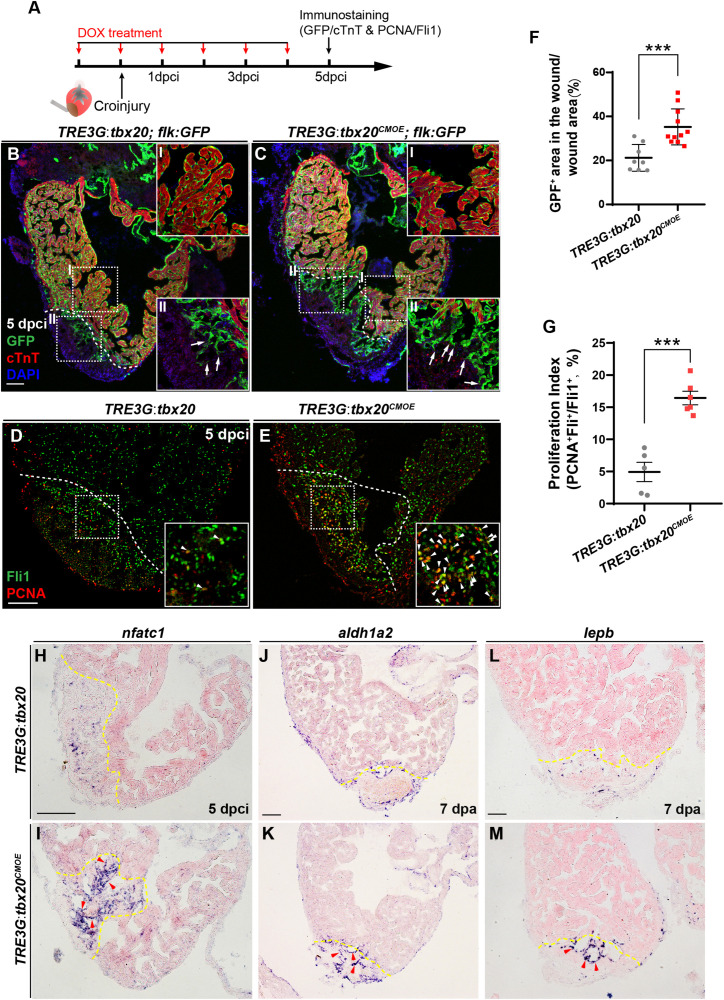
Myocardial *tbx20* overexpression enhances endocardial activation and regeneration. **(A)** Schematic of experimental procedures for DOX treatment, cryoinjury and immunostaining experiments. **(B,C)** Representative confocal fluorescence images of heart sections from DOX-treated *Tg*(*TRE3G:tbx20*; *flk:GFP*) **(B)** and *Tg*(*TRE3G:tbx20^*CMOE*^*; *flk:GFP*) **(C)** zebrafish at 5 dpci immunostained for cTnT (red), GFP and DAPI. Boxed areas (I and II) indicate locations of the magnified insets, respectively. Box-I in **(B,C)** indicate the remote (uninjured) region of heart sections, and Box-II in **(B,C)** display the endocardial cells in injured site. Arrows in **(B-II)** and **(C-II)** point to the endocardial cell protrusions. **(D,E)** Representative confocal fluorescence images of heart sections from DOX-treated *Tg*(*TRE3G:tbx20*) **(D)** and *Tg*(*TRE3G:tbx20^*CMOE*^*) fish **(E)** at 5 dpci immunostained for Fli1 (green) and PCNA (red). Boxed areas indicate locations of the magnified insets. Arrowheads point to the Fli1^+^PCNA^+^ proliferating endocardial/endothelial cells. **(F)** Scatter plot showing the percentage of GFP^+^ area within the wound area of DOX treated *Tg*(*TRE3G:tbx20*) (*n* = 8) and *Tg*(*TRE3G:tbx20^*CMOE*^*) (*n* = 11) hearts at 5 dpci. Mean ± SEM, ***p < 0.001. **(G)** Dotted diagram indicates the proliferation indices of endocardial/endothelial cells with Fli1^+^PCNA^+^ signals in the border zone and injury site from 5 dpci *Tg*(*TRE3G:tbx20*) (*n* = 5) and *Tg*(*TRE3G:tbx20^*CMOE*^*) (*n* = 6) cardiac sections. Mean ± SEM, ***p < 0.001. **(H–M)** Representative images of ISH analysis for *nfatc1* **(H,I)**, *aldh1a2* **(J,K)**, and *lepb* **(L,M)** expression at 5 dpci or 7 dpa hearts from DOX-treated *Tg*(*TRE3G:tbx20*) and *Tg*(*TRE3G:tbx20^*CMOE*^*) fish. Dashed lines delineate the injured area. Red arrowheads indicate endocardium with *nfatc1* **(I)**, *aldh1a2* **(K)** and *lepb* **(M)** signal in the injury site. Scale bar: 100 μm.

Previous studies revealed that there is a highly dynamic endocardium during cardiac regeneration, including changes in cell morphology, behavior and gene expression (Kikuchi et al., 2011b; Munch et al., 2017). As enhanced expression of *tbx20* in CMs augments heart regeneration after injury (Figure 2), we wondered if CM-specific *tbx20* overexpression also affects dynamics of endocardium in injured hearts. To test this possibility, we evaluated the impact of CM-specific *tbx20* overexpression on the behaviors of endocardium in injured hearts. Vascular endothelial growth receptor 2 (Vegfr2/Kdrl/Flk) and ETS-family transcriptional factor Fli1 are well-known endothelial/endocardial cell markers in the cardiovascular field and can be used for visualizing endocardial cell morphology and nuclei, respectively, during zebrafish heart regeneration (Munch et al., 2017; Sanchez-Iranzo et al., 2018; Zhao et al., 2019). *Tg*(*TRE3G:tbx20; flk:GFP*) and *Tg(TRE3G:tbx20^*CMOE*^; flk:GFP*) were generated by crossing the *Tg*(*flk:GFP*) with *Tg*(*TRE3G:tbx20)* line and *Tg*(*TRE3G:tbx20^*CMOE*^*) line, respectively. We damaged heart tissues using cryoinjury methods rather than ventricular apex resections, because the retained wound tissue following cryoinjury allows us to visualize the revascularization process and the dynamic of endocardium (Marin-Juez et al., 2016; Munch et al., 2017). DOX-treated *Tg(TRE3G:tbx20^*CMOE*^; flk:GFP*) animals and *Tg*(*TRE3G:tbx20; flk:GFP*) control fish were subjected to cryoinjury (Figure 5A). The injured hearts were immunostained with cTnT antibody that labels CMs and GFP antibody that recognizes *flk:GFP*-marked endocardial cells (Figure 5A). Previous studies report that endocardial expansion within the injury site is mediated through extension and migration of existing endocardial cells from the uninjured site (Munch et al., 2017). In the remote (uninjured) region of the *tbx20*-overexpressing and control heart, a coherent network of *flk:*GFP-marked endocardial cells surrounded cardiac muscles labeled by cTnT (Figures 5B,B-I,C,C-I). Within the injury site, we observed that a population of disorganized endocardial cells extended from the uninjured site and displayed cell protrusions, suggestive of migration (arrows in Figures 5B-II,C-II; Munch et al., 2017). Remarkably, Tbx20-overexprssion hearts in DOX-treated *Tg(TRE3G:tbx20^*CMOE*^; flk:GFP*) animals exhibited a marked increase in endocardial cells labeled by *flk:GFP* extending the injury site (Figures 5C,C-II). Quantification analyses indicated the proportion of the injured area occupied by *flk*:GFP^+^ cells was increased in *tbx20*-overexpressing hearts (35.2 ± 2.5%) compared to that in controls (21.2 ± 2.2%) (Figure 5F). These findings suggest that myocardial *tbx20* promotes extension and migration of endocardial cells into the injury site. We also appraised the endocardial cell proliferation adjacent to and within the cryoinjury site from DOX-treated *TRE3G:tbx20^*CMOE*^* hearts at 5 dpci by co-immunostaining for endothelial/endocardial cell nuclear marker Fli1 and cell proliferation marker PCNA (Figure 5A). We observed approximately threefold increase of proliferating endocardial/endothelial cells (Fli1^+^PCNA^+^) in*TRE3G:tbx20^*CMOE*^* hearts (16.4 ± 1.0%) in comparison with that of control hearts (4.9 ± 1.5%) (Figures 5D,E,G). Taken together, these findings indicate that myocardial-specific *tbx20* overexpression promotes endocardial cell migration and proliferation during heart regeneration.

Next, we assessed whether the endocardium is activated in myocardial *tbx20* overexpressing hearts. Nfatc1 is required for endocardial development in the heart and serves as a specific marker for endocardial activation after cardiac injury (Wong et al., 2012; Munch et al., 2017). ISH analyses revealed a striking upregulation of *nfatc1* at the injury site at 5 dpci in DOX-treated *TRE3G:tbx20^*CMOE*^* hearts (Figures 5H,I). In consistent, re-survey of our RNA-seq data revealed an increase of two other endocardial markers, *aldehyde dehydrogenase 1 family member a2* (*aldh1a2*) and *leptin b* (*lepb*), in DOX-treated *TRE3G:tbx20^*CMOE*^* hearts (Supplementary Table S3). Previous studies report that the Retinoic Acid synthesis enzyme Aldh1a2 and the secreted regulator of energy homeostasis protein Lepb are induced in the endocardium after cardiac injury, indicative of endocardial activation (Kikuchi et al., 2011b; Kang et al., 2016). We also validated the upregulation of *aldh1a2* and *lepb* at endocardial cells in the injured region in myocardial *tbx20* overexpressing hearts by ISH analyses (Figures 5J–M). Altogether, these results suggest that inducible *tbx20* overexpression in the adult myocardium contributes to endocardial cell migration and regeneration through endocardial cell activation, revealing crosstalk between the myocardium and endocardial cells during regeneration.

### Tbx20 Induction Augments Endocardial Bmp6 Signaling During Heart Regeneration

**FIGURE 6:**
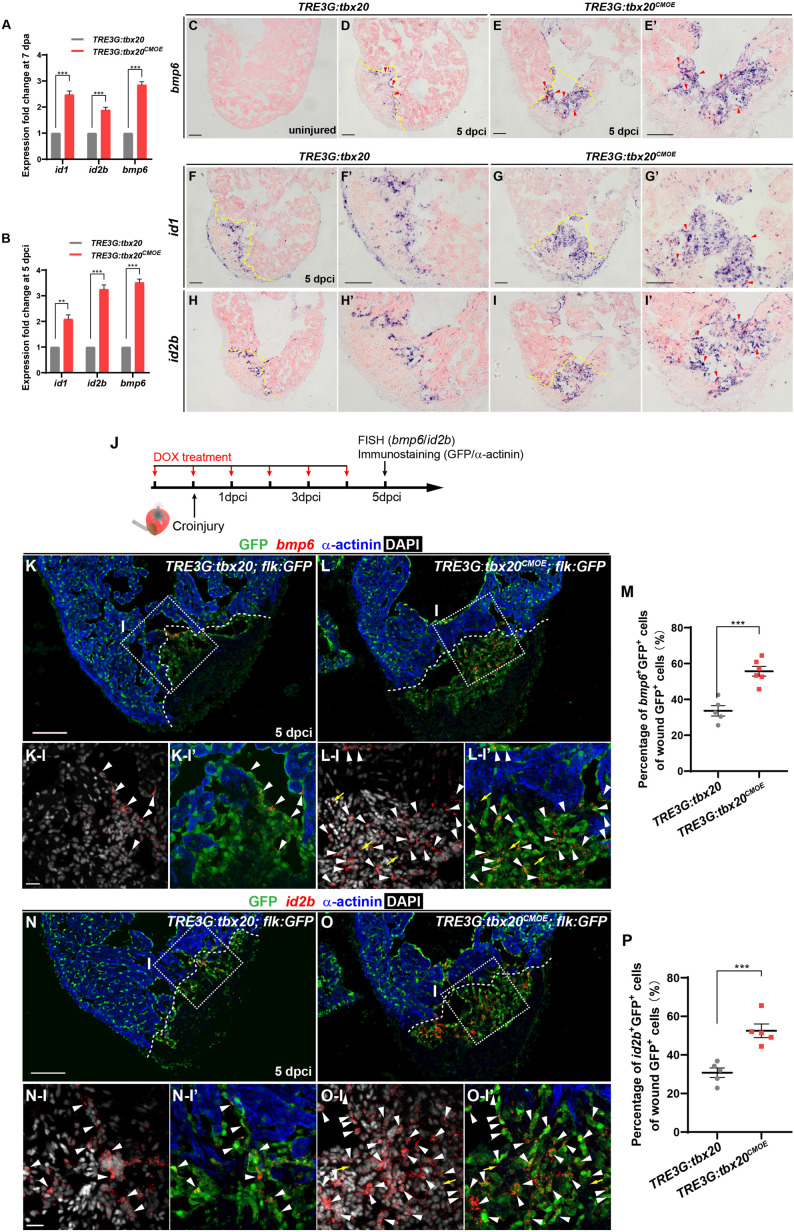
Myocardial Tbx20 mediates endocardial regeneration by activating Bmp6 signaling. **(A,B)** Myocardial *tbx20* overexpression upregulates BMP target genes *id1* and *id2b* as well as the BMP ligand *bmp6* after heart resection **(A)** or cryoinjury **(B)**, respectively. Data from three biological replicates. Mean ± SEM, **p < 0.01, ***p < 0.001. **(C–E)** Representative ISH images of heart sections from DOX-treated uninjured *Tg*(*TRE3G:tbx20*) **(C)**, 5 dpci *Tg*(*TRE3G:tbx20*) **(D)** and *Tg*(*TRE3G:tbx20^*CMOE*^*) zebrafish **(E,E’)** with *bmp6* probe. Red arrowheads indicate endocardium with *bmp6* signal. **(F–I)** Representative ISH images of heart sections from 5 dpci DOX treated *Tg*(*TRE3G:tbx20*) and *Tg*(*TRE3G:tbx20^*CMOE*^*) fish with *id1* **(F,G)** and *id2b* **(H,I)** probe. Red arrowheads indicate endocardium with *id1* **(G’)** and *id2b* **(I’)** signal in the injury site. **(J)** Experimental procedures for DOX treatment, cryoinjury, FISH combined with immunostaining experiments. **(K,L)** Representative images of FISH analysis of *bmp6* (red) combined with immunostaining for GFP and α-actinin (blue) on heart sections from 5 dpci *Tg*(*TRE3G:tbx20;flk:GFP*) **(K)** and *Tg*(*TRE3G:tbx20^*CMOE*^;flk:GFP*) **(L)** zebrafish. Boxed areas indicate locations of the magnified and channel-separated panels below. White arrowheads point to GFP^+^ cells with *bmp6* transcripts in border zone and injury site **(K-I,K-I’,L-I,L-I’)**, yellow arrows in **(L-I)** and **(L-I’)** indicate non-endocardial cells with *bmp6* transcripts. **(M)** Dotted diagram indicates the percentage of *bmp6*^+^ endocardial cells in injury site from 5 dpci DOX treated *Tg*(*TRE3G:tbx20*) (**K**, *n* = 5) and *Tg*(*TRE3G:tbx20^*CMOE*^*) (**L**, *n* = 6) fish. Mean ± SEM, ***p < 0.001. **(N,O)** Representative images of FISH analysis of *id2b* (red) combined with immunostaining for GFP and α-actinin (blue) on heart sections from 5 dpci *Tg*(*TRE3G:tbx20;flk:GFP*) **(N)** and *Tg*(*TRE3G:tbx20^*CMOE*^;flk:GFP*) zebrafish **(O)**. Boxed areas indicate locations of the magnified and channel-separated panels below. White arrowheads point to GFP^+^ cells with *id2b* transcripts in border zone and injury site **(N-I,N-I’,O-I,O-I’)**, yellow arrows in **(O-I)** and **(O-I’)** indicate non-endocardial cells with *id2b* transcripts. **(P)** Dotted diagram indicates the percentage of *id2b*^+^ endocardial cells in injury sites from 5 dpci DOX treated *Tg*(*TRE3G:tbx20*) (**N**, *n* = 5) and *Tg*(*TRE3G:tbx20^*CMOE*^*) (**O**, *n* = 5) fish. Mean ± SEM, ***p < 0.001. Dashed lines delineate the injured area. Scale bar: 100 μm **(C-I,K,L,N,O)**, 20 μm **(K-I-L-I’,N-I-O-I’)**.

To identify the molecular signaling involved in activating the endocardial regeneration program in the injured hearts with CM-specific *tbx20* overexpression, we searched differentially expressed genes participating in endocardial/endothelial activation in our RNA-seq/GO analysis data generated from control and *tbx20*-overexpressing hearts. We found a profound upregulation of BMP signaling, including *bmp6* ligand and its downstream targets, *inhibitor of DNA-binding proteins* (*id1*, *id2a* and *id2b*) in myocardial *tbx20* overexpressing hearts following ventricular injury (Figures 6A,B and Supplementary Table S3). ISH analyses revealed that the expression of *bmp6*, *id1* and *id2b* was marked induced in the injury border zone and the inside of the wound endocardium at 5 dpci hearts from DOX-treated *TRE3G: tbx20^*CMOE*^* fish in comparison to *TRE3G: tbx20* control fish (Figures 6C-I’). To determine the identity of *bmp6*^+^ cells in the injury site of hearts, we performed fluorescence *in situ* hybridization (FISH) experiments using *bmp6* or *id2b* antisense-mRNA probes on DOX-treated *Tg*(*TRE3G:tbx20^*CMOE*^;flk:GFP*) hearts and *Tg*(*TRE3G:tbx20;flk:GFP*) control hearts following cryoinjury (Figure 6J). These fluorescence hybridization hearts were subjected to double immunostaining of GFP antibody recognizing the *flk:GFP* transgenic endocardium and α-actinin antibody labeling cardiac muscle at 5dpi (Figure 6J). We found that *bmp6* transcripts were located predominantly in *flk:GFP*-marked endocardial cells in *tbx20*-overexpressing and control hearts (arrowheads in Figures 6K,K-I,K-I’,L,L-I,L-I’); however, only a few number of non-endocardial cells expressed *bmp6* in *tbx20*-overexpressing hearts (arrows in Figures 6L-I,L-I’). Quantification analyses indicated a significant increase of *bmp6*^+^Flk-GFP^+^ cells in *tbx20*-overexpressing hearts (55.7 ± 2.7%), in comparison with control hearts (33.6 ± 2.9%) (Figure 6M). Similarly, expression of *id2b*, a Bmp6 downstream target, overlapped with *flk*:GFP in endocardial cells in both DOX-treated *TRE3G:tbx20^*CMO*^*^*E*^ hearts (arrowheads in Figures 6O,O-I,O-I’) and *TRE3G:tbx20* control hearts (arrowheads in Figures 6N,N-I,N-I’). *id2b* transcripts were only detectable in a few non-endocardial cells in *tbx20*-overexpressing hearts (arrows in Figures 6O-I,O-I’). Importantly, myocardial *tbx20* overexpression resulted in a significant increase in *id2b*^+^Flk-GFP^+^ endocardial cells at the cardiac injury region (52.5 ± 3.5%) compared to that in *TRE3G:tbx20* control hearts (30.2 ± 2.5%) (Figure 6P). In contrast, expression of *bmp3* or *bmp10* was not significantly upregulated in *tbx20* overexpressing wound hearts (Supplementary Figure S8). Collectively, these findings indicated that Bmp6/Id2b signaling is activated mostly in the endocardium in response to Tbx20 induction following cardiac injury.

**FIGURE 7:**
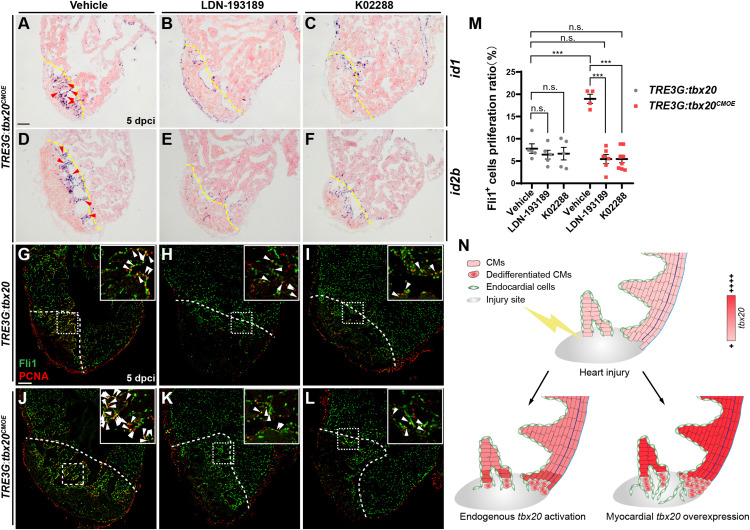
Inhibition of Bmp6 signaling restricts endocardial cell proliferation activated by myocardial *tbx20* overexpression. **(A–F)** Representative ISH images showing expression of *id1* **(A–C)** and *id2b* **(D–F)** in DOX treated *Tg*(*TRE3G:tbx20^*CMOE*^*) fish at 5 dpci after vehicles or inhibitors treatment. Red arrowheads indicate endocardium with *id1* **(A)** and *id2b* **(D)** signal in the injury site. **(G–L)** Representative confocal fluorescence images of heart sections immunostained for Fli1 (green) and PCNA (red) from vehicle, LDN-193189 and K02288 treated control **(G–I)** and myocardial *tbx20* overexpressing fish **(J–L)** at 5 dpci. Boxed areas indicate locations of the magnified insets. Arrowheads point to the Fli1^+^PCNA^+^ proliferating endocardial cells. **(M)** Scatter plot showing the percentage of Fli1^+^ cell proliferation ratio in the border zone and injury site from **(G–L)**. The values are mean ± S.E.M. Two-way ANOVA followed with Tukey’s multiple comparison test. n.s.: none significance, ***p < 0.001. **(N)** Model of myocardial *tbx20* function during zebrafish heart regeneration: In CMs, myocardial *tbx20* overexpression promotes CM dedifferentiation and proliferation following injury. In addition, myocardial *tbx20* promotes endocardial regenerative progress by enhancing its proliferation via partially through Bmp6 signaling. Dashed lines delineate the injured area. Scale bar: 100 μm.

To test whether the increase of endocardial regeneration caused by myocardial *tbx20* overexpression is Bmp6 signaling-dependent, two highly specific BMP signaling inhibitors, LDN-193189 and K02288, which selectively interferes with BMP type I Alk2, Alk3 receptors (Sanvitale et al., 2013; Palencia-Desai et al., 2015), were used to assess the effect of Bmp6 signaling on Tbx20-mediated endocardial regeneration. LDN-193189 or K02288 was intraperitoneally injected every 24 h starting at 2 dpci. We found that BMP inhibitor-treatments diminished *id1* and *id2b* expression at cryoinjury sites in DOX-treated *TRE3G:tbx20^*CMOE*^* animals (Figures 7A–F) but not *TRE3G:tbx20* control animals (Supplementary Figure S9), suggesting that upregulation of Bmp6 signaling is Tbx20-dependent following injury. Moreover, co-immunostaining with anti-PCNA and anti-Fli1 antibodies revealed that endocardial cell proliferation adjacent to and within the cryoinjury site in LDN-193189- or K02288-treated *TRE3G:tbx20* control hearts were comparable to that in vehicle-treated control hearts (vehicle: 7.8 ± 1.1%; LDN-193189: 6.4 ± 1.0%; K02288: 6.7 ± 1.4%) (Figures 7G-I,M). On the contrary, the increased endocardial cell proliferation was significantly suppressed (vehicle: 19.0 ± 1.0%; LDN-193189: 5.5 ± 1.0%; K02288: 5.5 ± 0.8%) in wounded *TRE3G:tbx20^*CMOE*^* hearts treated with LDN-193189 or K02288 inhibitors (Figures 7J–L,M). These findings suggest that CM-specific *tbx20* overexpression induces endocardial cell proliferation, at least in part, by upregulating Bmp6 signaling.

## Discussion

Based on our findings, we propose a working model for Tbx20-dependent transcriptional network in governing heart regeneration using zebrafish heart injury model (Figure 7N). Following heart injury, *tbx20* is strongly induced in the myocardial wound region and the atrial epicardium. Myocardial-induced *tbx20* upregulates expression of embryonic or fetal genes as well as cell-cycle regulators, promoting CM proliferation via dedifferentiation. We find that Tbx20 induction also plays a key role in endocardial cell migration and regeneration through upregulating endocardial Bmp6 signaling after cardiac damage. Thus, this putative Tbx20-mediated transcriptional program would coordinate two distinct mechanisms of zebrafish heart regeneration.

Because adult hearts are least likely to exist cardiac stem cells (Kretzschmar et al., 2018; He et al., 2019), myocardial regeneration occurs via stem-cell independent mechanisms, in which resident CMs in the injured heart undergo dedifferentiation prior to their proliferation (Jopling et al., 2010; Kikuchi et al., 2010; Senyo et al., 2013). Our study indicates that inducible *tbx20* overexpression in adult zebrafish hearts enhance CM proliferation and myocardial regeneration, similar to that observed in mice by Xiang et al. (2016). Mechanistically, we have demonstrated that Tbx20 promotes injury-induced CM proliferation via dedifferentiation through mediating cellular changes and molecular dedifferentiation circuits. Specifically, CMs at the wound border zone express a myocardial fetal marker α-SMA and a stem cell marker Runx1 in injured zebrafish hearts. Both α-SMA and Runx1 were discovered originally as CM dedifferentiation markers in human patients suffered from myocardial infarction (Kubin et al., 2011). Thus, CM dedifferentiation following cardiac injury is a conserved mechanism, defining zebrafish as a model system to study injury-induced CM dedifferentiation during regeneration. Interestingly, we find that *tbx20* induction is not only localized to the injured myocardium, but also detectable in the atrial epicardium following ventricle injury. During development, *tbx20* is expressed in epicardial cells surrounding the atrioventricular canal groove (Yamagishi et al., 2004; Boogerd et al., 2018), but the function of epicardial *tbx20* is still unclear. Previous studies indicate that epicardium is required for heart regeneration in zebrafish (Kikuchi et al., 2011a; Wang et al., 2015). It will be important to test whether atrial *tbx20*^+^ epicardial cells contribute to injury-induced CM proliferation, and how atrial epicardial activation relates to ventricle regeneration.

BMP signaling plays pivotal functions via diverse mechanisms involving vascular, myocardial, endocardial and mesenchymal tissues during cardiovascular development (Morrell et al., 2016). We determined that Bmp6 signaling in the endocardium is an early injury-response to myocardial Tbx20 induction, which promotes endocardial cell regeneration. Precedent for this type of crosstalk between myocardium and endocardium exists in the context during heart development. For example, Bmp2 and Bmp4 function in the myocardium is required for the epithelial-mesenchymal transformation (EMT) within the endocardium, leading to the formation of endocardial cushions and valves (Ma et al., 2005; Choi et al., 2007). During zebrafish heart regeneration, our RNA-seq analyses revealed upregulation of multiple *bmp* ligands in *tbx20*-overexpressing hearts, consistent with part of BMP regulation following cardiac injury (Wu et al., 2016). However, the cellular sources of secreted *bmp* ligands remain unclear in injured hearts. In this study, we determined that *bmp6* and *id2b* are primarily upregulated in the endocardium following diverse cardiac damages, while their expression was not detectable in uninjured WT hearts, revealing Bmp6 as a specific BMP signal in the endocardium in response to cardiac injury. Previous studies report that Notch signaling and Retinoic Acid (RA) pathways are activated in the wound endocardium (Kikuchi et al., 2011b; Munch et al., 2017). However, both Notch and RA pathways in the endocardium signal to the myocardium to stimulate CM regeneration (Kikuchi et al., 2011b; Munch et al., 2017; Zhao et al., 2019). Our data suggest that Bmp6 signaling in the endocardium promotes endocardial cell regeneration in response to myocardial Tbx20 induction. In uninjured mouse hearts, Tbx20-mediated increases in CM proliferation are due to activation of Bmp2 and PI3K signaling in the myocardium (Chakraborty et al., 2013). Thus, the influence of Tbx20 on distinct Bmp ligands in myocardial cells or the endocardium appears to be context dependent. We have also observed the increased immune response genes in Tbx20-overexpressing hearts in RNA-seq analyses. Immune response participates in heart injury and repair (Lai et al., 2019). Previous studies report that immune cells, such as macrophages, play important roles in the process of inflammation, scar resolution and wound healing, which in turn stimulates myocardial regeneration and repair (Lai et al., 2019). It will be interesting to test whether Tbx20 directly or indirectly regulates immune response genes during cardiac regeneration, and whether Bmp6/Id2b signaling might be activated in immune cells such as macrophages following cardiac injury.

Although adult zebrafish can efficiently regenerate their hearts in response to different forms of damage, there are some intrinsic disadvantages to the model system. For instances, proliferative CMs in zebrafish are mononuclear and diploid, whereas most adult human CMs become polyploidy that restrains CM proliferation (Xin et al., 2013; Tzahor and Poss, 2017). Although CM renewal is extremely low in uninjured mammalian hearts, a small number of proliferative CMs can be detectable in the injury border zone of murine hearts after myocardial infarction (Malliaras et al., 2013; Xiang et al., 2016; Xie et al., 2020). We believe that specific factors and mechanisms obtained from zebrafish regeneration study should be confirmed in mammal system such as mouse before exploring potential therapeutics in humans. Tbx20 plays a conserved role in promoting injury-induced CM regeneration in zebrafish and mouse (Xiang et al., 2016), raising a possibility that Tbx20 might be a target to be explored in the medical context. For instance, Tbx20 administration at local injury regions might be potential strategy to improve repair capacity in the wound human heart. Overall, understanding of the mechanisms underlying heart regeneration in model systems will provide inspiration for the regeneration intervention in humans.

## Materials and Methods

### Zebrafish Cardiac Injury

All animal work used in this study were approved by the Animal Care Committee of Fudan University. We bred wild-type zebrafish, or zebrafish carrying *Tg*(*flk:GFP*) and *Tg*(*cmlc2:GFP*) (Burns et al., 2005; Jin et al., 2005). In addition, we bred zebrafish carrying the newly generated transgenes described below. 6–18 months aged wild type or transgenic lines of the AB strain were used for ventricular resection or cryoinjury as previously described (Poss et al., 2002; Gonzalez-Rosa et al., 2011). Tricaine (ethyl-3-aminobenzoate methane sulfonate salt, Sigma) with concentration of 0.16 mg/mL were used to anesthetize zebrafish before use.

### Generation of *Tg*(*tbx20:moxGFP*), *Tg*(*cryaa:mCherry*; *cmlc2:TetON-3G*) and Tg(*cryaa:EGFP*; *TRE3G:tbx20-E2A-mCherry*) Zebrafish

A 7.3-kb promoter of *tbx20* was amplified from zebrafish genome and cloned upstream of *moxGFP* in plasmid pT2KXIGΔin plasmid to replace *xenopus ef1*α to *EGFP* (Costantini et al., 2015). To generate the transgenic line, *Tol2* mRNA and the plasmid were co-injection into one-cell stage fertilized eggs.

The *cmlc2*-TetON-3G cassette was first constructed by cloning TetON-3G from pCMV-Tet3G vector (Clontech, #z1164n) downstream of a 3.0-kb *cmlc2* promoter. In order to identify the transgenic animals by lens fluorescence, the *Cryaa*-mCherry cassette from plasmid pT2-hsp70l-dnMEK1 (Liu and Zhong, 2017) was then cloned downstream of the *cmlc2*-TetON-3G cassette in the opposite orientation. The entire construct was franked by two copies of the chicken β-globin insulators at upstream and one human 5′β-globin insulator at downstream, and one human 3′β-globin insulator was also included between the two cassettes.

The *TRE3G*-tbx20-E2A-mCherry cassette was first constructed by cloning TRE3G sequence from pTRE3G-Bl vector (Clontech, #z1164n) upstream of the assembled tbx20-E2A-mCherry fusion gene. mCherry fragment in *Cryaa*-mCherry cassette described above was replaced by EGFP using NEB HIFI Assembly Master Mix (NEB, #E2621S). *Cryaa*-EGFP cassette was then cloned downstream of the *TRE3G*-tbx20-E2A-mCherry cassette in the opposite orientation. The entire construct was insulated as same as *cryaa:mCherry;cmlc2:TetON-3G* (see above paragraph). These constructs were injected into one-cell-staged embryos, respectively, using Tol2 transposase-meditated transgenesis techniques (Suster et al., 2009).

### Immunostaining, ISH, AFOG and Histology Analyses

Zebrafish hearts were dissected and fixed in 4% paraformaldehyde (PFA) solution at 4% overnight before embedded in OCT (Thermo Fisher Scientific). 10 μm cryosections were used in all histological analyses, *in situ* hybridization (ISH) and immunostaining. The primers used to generate RNA probes were listed in Supplementary Table S4. ISH and Acid Fushin-Orange G (AFOG) analyses were performed as described, the quantification of the scar area was performed using Fiji software (Poss et al., 2002). The TSA -Plus fluorescence system (Perkin Elmer, #NEL752001KT) were used for fluorescence *in situ* hybridization (FISH) analyses according to the manufacturer’s instructions. Primary and secondary antibody staining were performed according to the standard protocol, except for PCNA staining which required heat-induced antigen retrieval (Lepilina et al., 2006). All primary antibodies were incubated at 4% overnight and secondary antibodies were incubated at room temperature for 2 hrs. Primary antibodies used in this study include GFP (Invitrogen, #A21311, 1:200), GFP (Aves Labs, #GFP-1010, 1:500), α-actinin (Sigma-Aldrich, #A7732, 1:250), PCK (Abcam, #ab86734, 1:200), PCNA (Sigma-Aldrich, #P8825, 1:300), Mef2 (Santa Cruz Biotechnology, #sc-313, 1:75), ZO-1 (Invitrogen, #339188, 1:200), α-SMA (Genetex, #GTX124505, 1:250), cTnT (DSHB, #CT3,1:200), N-cadherin (Abcam, #76011, 1:250), Runx1 (Abcam, #ab92336, 1:250) and Fli1 (Abcam, #ab133485, 1:250). Secondary antibodies used in this study include goat anti-mouse Alexa Fluor 647 (Invitrogen, #A21236), goat anti-mouse Alexa Flur 488 (Invitrogen, #A28175), goat anti-rabbit Alexa Fluor 594 (Invitrogen, #A11037), goat anti-rabbit Alexa Fluor 568 (Invitrogen, #A11011), goat anti-chicken Alexa Fluor 488 (Invitrogen, #A-11039) and goat anti-rabbit Alexa Fluor 488 (Invitrogen, #A11034) with concentration of 1:1000.

AFOG images were taken using an Olympus DP80 microscope. ISH images were taken using a Nikon microscope. FISH and immunofluorescence images were taken using a ZEISS LSM880 confocal microscope. For quantitative analyses of immunofluorescence images, sections containing the largest wounds were selected and manually counted. A defined region within and adjacent (150 μm and 100 μm away from the wound edge for Mef2^+^PCNA^+^ CMs and Fli1^+^PCNA^+^ cells, respectively) to the injury site were selected to quantify the proliferation ratio of CMs and endothelial cells.

### Gene Expression Analysis

For RNA-seq analysis, 7 dpa hearts were dissected, and the apical portions of 6-8 ventricles were collected in each group. RNA samples were extracted using TRIzol Reagent (Invitrogen, #15596018). Next generation sequencing library construction and sequencing was performed by GENEWIZ Company, Suzhou on an Illumina HiSeq sequencer. Reads from the sequenced samples were qualified and aligned to zebrafish transcriptome (Ensembl genebuild GRCz10.86) using Hisat2 (v2.0.1). Differentially expressed genes were analyzed using the DESeq Bioconductor package, genes identified with altered expression levels with a Benjamini and Hochberg adjusted *p*-value < 0.05 were retained. GO-TermFinder was used to define Gene Ontology terms. For quantitative RT-PCR analysis (qPCR), RNA samples were extracted from ventricular apices, border zone and atrium at specific time points, respectively, 6-8 samples were pooled together for each group, cDNA was synthesized using the SuperScript^TM^ III First-Strand Synthesis System (Invitrogen, #18080051) following the manufacturer’s instructions. q-PCR was performed on a Q7 Real-Time PCR System (Applied Biosystems) using SYBR Green ROX dye (Applied Biosystems, #A25742). Primers used are listed in Supplementary Table S4.

### Small Molecule Treatments

Doxycycline (DOX) (Sigma-Aldrich, #d9891) was dissolved in ddH_2_O at 50mg/ml as stock solution. Adult fish were daily treated with 50 mg/L DOX from the day before heart injury. LDN-193189 (Selleck, #S7507) was dissolved in PBS to a final concentration of 50 mM, K02288 (Selleck, #S7359) was dissolved in DMSO to a final concentration of 50 mM. Adult zebrafish was injected intraperitoneally every 24 h with 10 μL LDN-193189 (50 μM in PBS), K02288 (50 μM in PBS) or vehicle (0.1% DMSO in PBS) from 2 dpci to 4 dpci.

### Statistical Analysis

All quantitative analyses of immunofluorescence images and assessment of stained images were performed in blinded fashion. GraphPad Prism 8 was used for data analyzing. Statistical comparisons between two groups were analyzed by Student’s *t*-test. For samples with more than two independent groups, one-way ANOVA with Dunnett’s multiple comparisons test were performed. For samples with two experimental factors, two-way ANOVA followed by Tukey’s multiple comparisons tests were performed. All statistical tests were calculated when normality test using D’Agostino-Pearson omnibus test and Shapiro-Wilk test was passed.

## Data Availability Statement

The datasets presented in this study can be found in online repositories. The names of the repository/repositories and accession number(s) can be found below: https://www.ncbi.nlm.nih.gov/geo/, GSE144831.

## Ethics Statement

The animal study was reviewed and approved by the Animal Care Committee of Fudan University.

## Author Contributions

YF, KL, and TZ designed the study. YF and KL performed the experiments. YF, KL, PS, JS, WT, and TZ analyzed the experimental data. YF and TZ wrote the manuscript. WT and TZ edited the manuscript. All authors contributed to the article and approved the submitted version.

## Conflict of Interest

The authors declare that the research was conducted in the absence of any commercial or financial relationships that could be construed as a potential conflict of interest.

## Supplementary Material

FIGURE S1 *tbx20* is upregulated in the injured hearts at 7 dpa and 5 dpci. **(A)** qPCR analyses of relative expression levels of *tbx20* in border zone and atrium from uninjured hearts and 1 dpa, 3 dpa and 7 dpa injured hearts. Data represent three biological replicates. Mean ± SEM, ^∗^*p* < 0.05, ^∗∗^*p* < 0.01, ^∗∗∗^*p* < 0.001. **(B–E)** Representative ISH images showing *tbx20* expression in uninjured and 5 dpci ventricles **(B,C)** and atriums **(D,E)**. **(F–M)** Representative images of ISH with *tbx20* sense probe on the heart sections of uninjured **(F,G)** and injured ventricles and atriums at indicated time points **(H–M)**. **(N–Q)** Representative confocal images of sections from ventricles **(N,O)** or atriums **(P,Q)** of uninjured and 5 dpci *Tg*(*tbx20:moxGFP*) hearts co-immunostaiend with GFP and α-actinin (red), or with GFP and PCK (red), respectively. Red arrowheads point to atrial epicardium. Dotted line demarcates the edge of the wound area. Scale bar: 100 μm.

FIGURE S2 *tbx20* is upregulated in atrial epicardium after injury, whereas not detectable in ventricular epicardium. **(A,B)** Single confocal plane of heart sections from uninjured **(A)** and 7 dpa **(B)** *Tg*(*tbx20:moxGFP*) hearts immunostained for GFP, PCK (red) and DAPI. **(A-I–A-II”), (B-I–B-II”)** are boxed regions in **(A)** and **(B)** showing split channels in a higher magnification, respectively. Scale bar: 100 μm **(A,B)**, 10 μm (magnified images).

FIGURE S3 No leakiness of *tbx20* expression is observed with DOX treatment using the TetON-3G system. **(A–D)** Adult transgenic zebrafish images indicating the selectable marker of lens, *Tg*(*TRE3G:tbx20*) showed green eyes in **(B)**, *Tg*(*TRE3G:tbx20^*CMOE*^*) showed yellow eyes in **(D)**. No mCherry signal was observed in the zebrafish fin, skin or muscle after DOX treatment **(D)**. Scale bar: 250 μm. **(E,F)** Images of whole mount hearts of the transgenic fish at 7 dpa treated with DOX in the brightfield **(E)** or mCherry channel **(F)**, mCherry signal was not detected in the outflow tract (asterisk) and the injured site (arrow) from *Tg*(*TRE3G:tbx20^*CMOE*^*) hearts. Scale bar: 500 μm. **(G–I)** ISH analysis of *tbx20* overexpression using *tbx20-E2A-mCherry* probe on 7 dpa heart sections from *Tg*(*TRE3G:tbx20*) zebrafish treated with DOX **(G)**, *Tg*(*TRE3G:tbx20^*CMOE*^*) zebrafish treated with **(I)** or without DOX solution **(H)**. Scale bar: 100 μm.

FIGURE S4 Proliferative CMs are almost not detectable in uninjured adult hearts with myocardial *tbx20* overexpression. **(A,B)** Confocal fluorescence images of uninjured adult heart sections from *Tg*(*TRE3G:tbx20*) **(A)** and *Tg*(*TRE3G:tbx20^*CMOE*^*) zebrafish **(B)** after 7 days of DOX treatment co-stained with PCNA (red) and Mef2 (green) antibody. Scale bar: 100 μm.

FIGURE S5 Myocardial *tbx20* overexpression resulted in loss of cell-cell contact in the border zone myocardium. **(A,B)** Representative confocal fluorescence images of injured ventricle sections from *Tg*(*TRE3G:tbx20*) **(A)** and *Tg*(*TRE3G:tbx20^*CMOE*^*) **(B)** zebrafish co-stained with antibodies against cTnT (green) and N-cadherin (red). DAPI was used to stain nuclei. Boxed areas are locations of magnified images in Figures 3C,D. Scale bar: 100 μm.

FIGURE S6 Myocardial *tbx20* overexpression promotes injury-induced α-SMA in CMs at border zone and compact layer. **(A)** Representative confocal fluorescence image of injured ventricle sections from *Tg*(*TRE3G:tbx20^*CMOE*^*) zebrafish co-stained with antibodies against α-SMA (red) and α-actinin (green). DAPI was used to stain nuclei. Boxed areas are magnified below with split channels. White arrows point to compact layer myocardium, white arrowheads indicate α-SMA^+^ CMs in regenerating trabecular layer, light blue arrows point to non-cardiomyocyte cells. Scale bar: 100 μm **(A)**; 20 μm **(A-I–A-II”)**.

FIGURE S7 Myocardial *tbx20* overexpression augments CMs dedifferentiation following injury. **(A–H)** Representative ISH images showing expression of *nppb* **(A,B)**, *gata4* **(C,D)**, *gata5* **(E,F)** and *hand2* **(G,H)** in the adult injured *Tg*(*TRE3G:tbx20*) and *Tg*(*TRE3G:tbx20^*CMOE*^*) zebrafish ventricles after DOX-treatment at 7 dpa. Dotted lines demarcate amputation plane. Sale bar: 100 μm. **(I)** Statistical analyses of qPCR for *gata4*, *gata5* and *hand2* in the injured ventricle apices from *Tg*(*TRE3G:tbx20*) and *Tg*(*TRE3G:tbx20^*CMOE*^*) zebrafish at 7 dpa. Data represent three biological replicates. Mean ± SEM, ^∗^*p* < 0.05, ^∗∗∗^*p* < 0.001.

FIGURE S8 Expression profile of different BMP ligands in injured hearts. **(A)** Heat map showing fold changes for transcripts of BMP signaling ligands in DOX-treated *Tg*(*TRE3G:tbx20*) and *Tg*(*TRE3G:tbx20^*CMOE*^*) at 7 dpa detected by RNA-seq. **(B–G)** ISH analyses for *bmp3* **(F,G)**, *bmp6* **(B,C)** and *bmp10* **(D,E)** on heart sections from DOX-treated *Tg*(*TRE3G:tbx20*) or *Tg*(*TRE3G:tbx20^*CMOE*^*) zebrafish at 7 dpa and 5 dpci. Red arrowheads in **(B,C)** indicate endocardium with *bmp6* signal. Dotted lines demarcate amputation plane. Sale bar: 100 μm.

FIGURE S9 *id1* and *id2b* expression are not altered in injured control hearts after BMP inhibitors treatment. **(A–F)** Representative ISH images showing expression pattern of *id1* **(A–C)** and *id2b* **(D–F)** in DOX treated *Tg*(*TRE3G:tbx20*) fish at 5 dpci after vehicle or inhibitors treatment. Dotted lines demarcate amputation plane. Scale bar: 100 μm.

TABLE S1 Differentially expressed genes in myocardial *tbx20* overexpressing ventricular apices compared with control at 7 days post amputation.

TABLE S2 Gene Ontology analysis of differentially expressed genes in *Tg*(*TRE3G:tbx20^*CMOE*^*) zebrafish hearts at 7 dpa.

TABLE S3 FPKM values of differentially expressed genes in control and myocardial *tbx20* overexpressing hearts at 7 dpa.

TABLE S4 Primers used in this study.
