# Supplementary figures and images for: Tbx20 Induction Promotes Zebrafish Heart Regeneration by Inducing Cardiomyocyte Dedifferentiation and Endocardial Expansion
Source: Front Cell Dev Biol. 2020 Aug 4;8:738. doi: 10.3389/fcell.2020.00738 (PMC7417483; doi:10.3389/fcell.2020.00738)

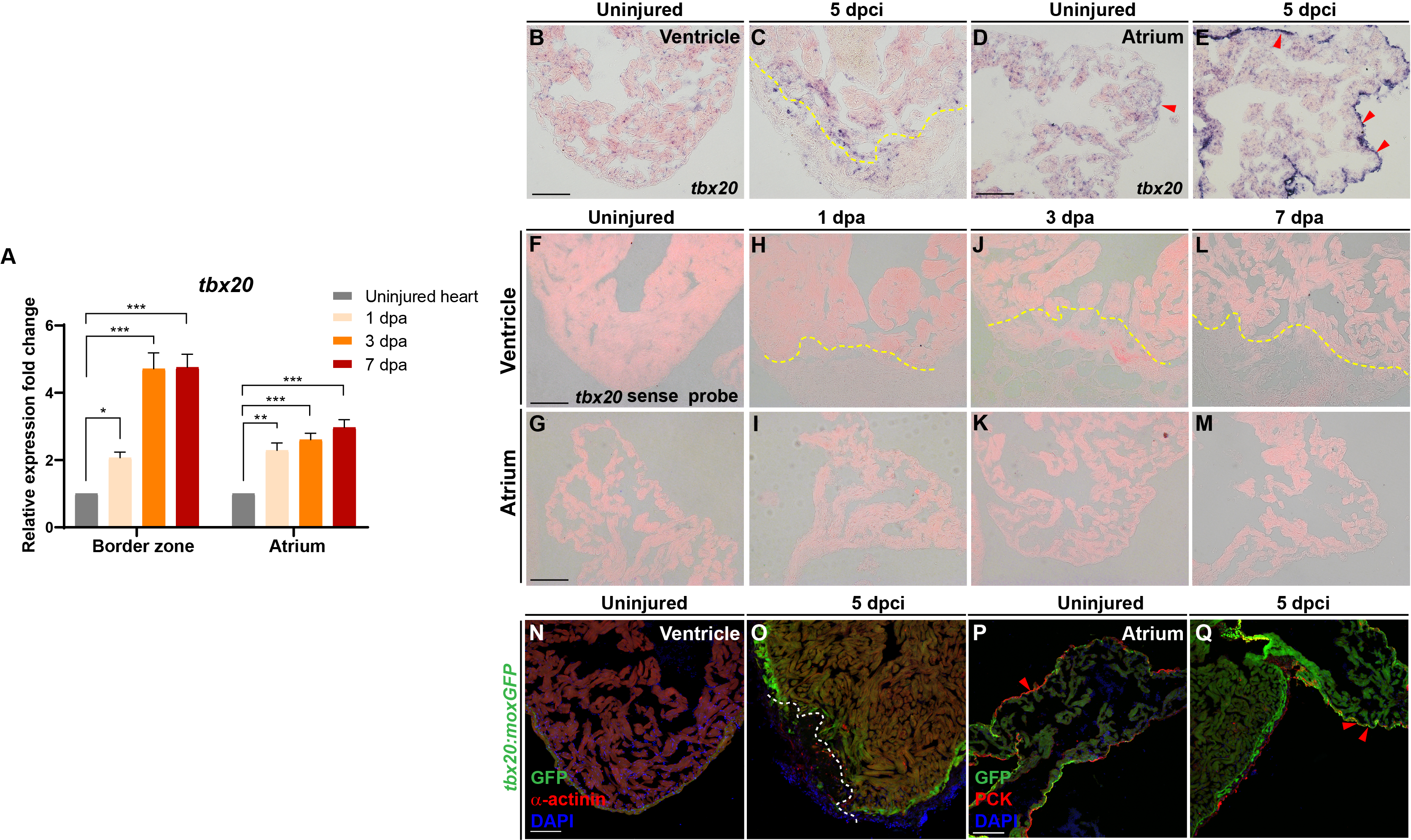

Supplement: FIGURE S1 — tbx20 is upregulated in the injured hearts at 7 dpa and 5 dpci. (A) qPCR analyses of relative expression levels of tbx20 in border zone and atrium from uninjured hearts and 1 dpa, 3 dpa and 7 dpa injured hearts. Data represent three biological replicates. Mean ± SEM, ∗p < 0.05, ∗∗p < 0.01, ∗∗∗p < 0.001. (B–E) Representative ISH images showing tbx20 expression in uninjured and 5 dpci ventricles (B,C) and atriums (D,E). (F–M) Representative images of ISH with tbx20 sense probe on the heart sections of uninjured (F,G) and injured ventricles and atriums at indicated time points (H–M). (N–Q) Representative confocal images of sections from ventricles (N,O) or atriums (P,Q) of uninjured and 5 dpci Tg(tbx20:moxGFP) hearts co-immunostaiend with GFP and α-actinin (red), or with GFP and PCK (red), respectively. Red arrowheads point to atrial epicardium. Dotted line demarcates the edge of the wound area. Scale bar: 100 μm. [file Image_1.TIF]

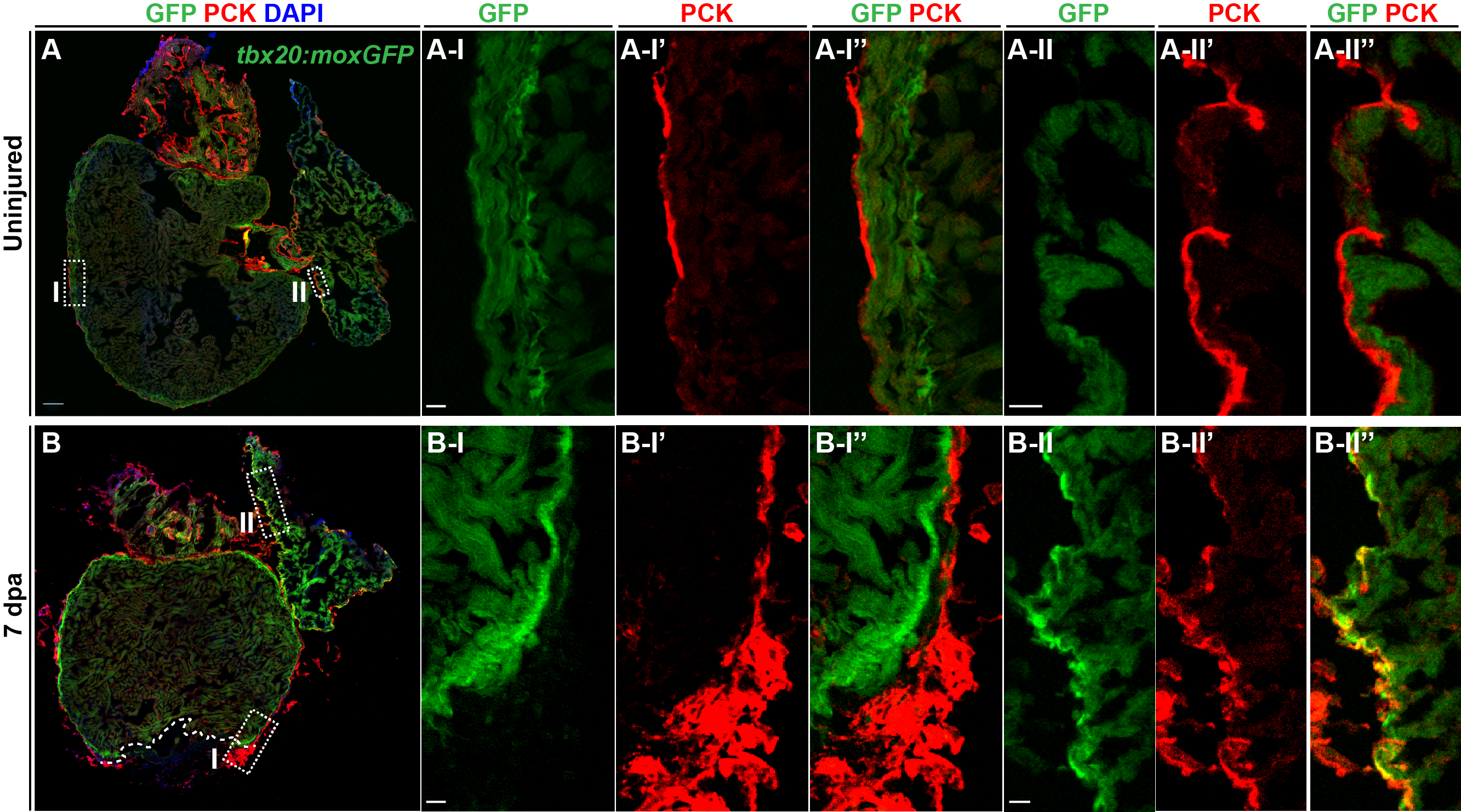

Supplement: FIGURE S2 — tbx20 is upregulated in atrial epicardium after injury, whereas not detectable in ventricular epicardium. (A,B) Single confocal plane of heart sections from uninjured (A) and 7 dpa (B) Tg(tbx20:moxGFP) hearts immunostained for GFP, PCK (red) and DAPI. (A-I–A-II”), (B-I–B-II”) are boxed regions in (A) and (B) showing split channels in a higher magnification, respectively. Scale bar: 100 μm (A,B), 10 μm (magnified images). [file Image_2.TIF]

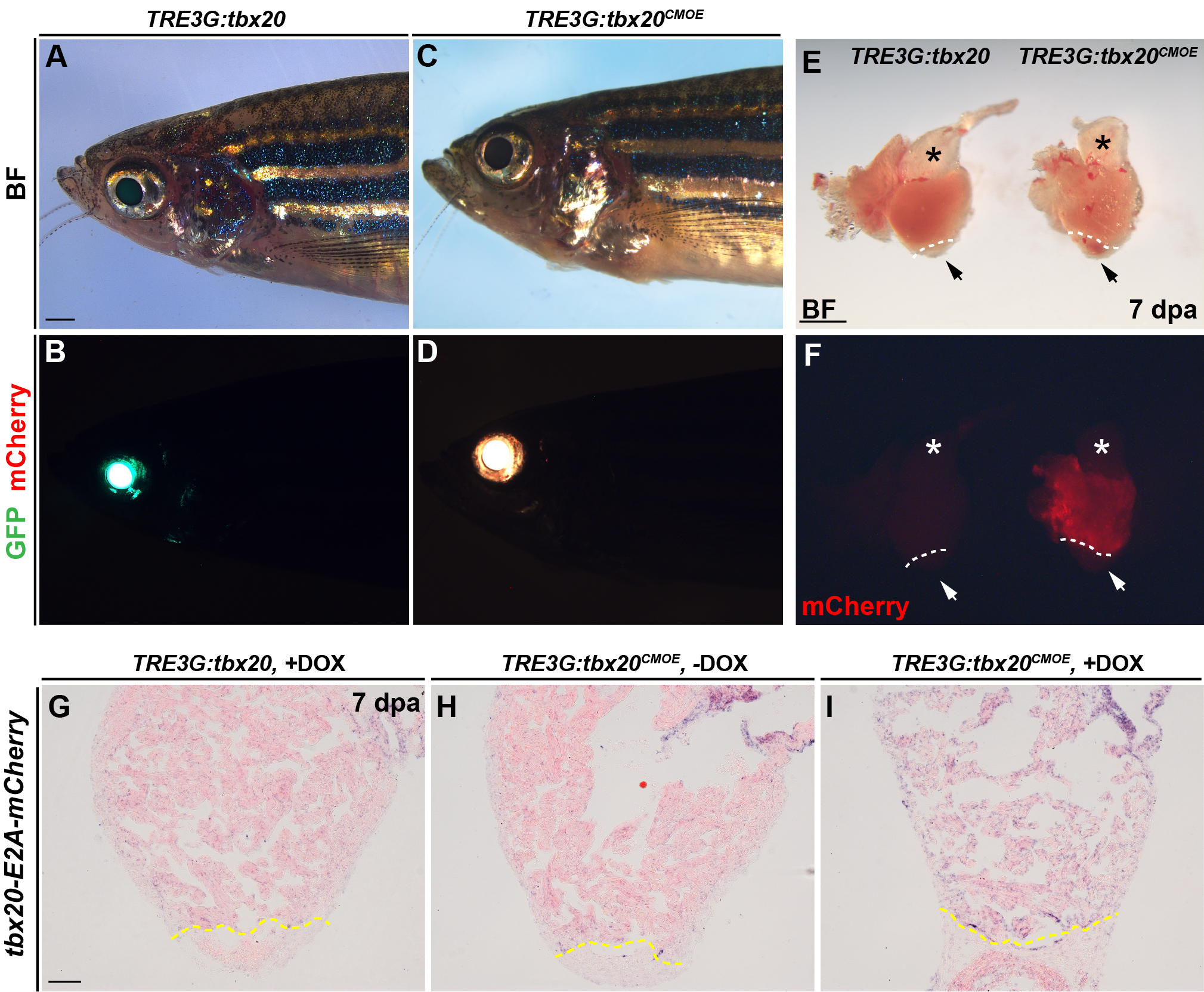

Supplement: FIGURE S3 — No leakiness of tbx20 expression is observed with DOX treatment using the TetON-3G system. (A–D) Adult transgenic zebrafish images indicating the selectable marker of lens, Tg(TRE3G:tbx20) showed green eyes in (B), Tg(TRE3G:tbx20CMOE) showed yellow eyes in (D). No mCherry signal was observed in the zebrafish fin, skin or muscle after DOX treatment (D). Scale bar: 250 μm. (E,F) Images of whole mount hearts of the transgenic fish at 7 dpa treated with DOX in the brightfield (E) or mCherry channel (F), mCherry signal was not detected in the outflow tract (asterisk) and the injured site (arrow) from Tg(TRE3G:tbx20CMOE) hearts. Scale bar: 500 μm. (G–I) ISH analysis of tbx20 overexpression using tbx20-E2A-mCherry probe on 7 dpa heart sections from Tg(TRE3G:tbx20) zebrafish treated with DOX (G), Tg(TRE3G:tbx20CMOE) zebrafish treated with (I) or without DOX solution (H). Scale bar: 100 μm. [file Image_3.TIF]

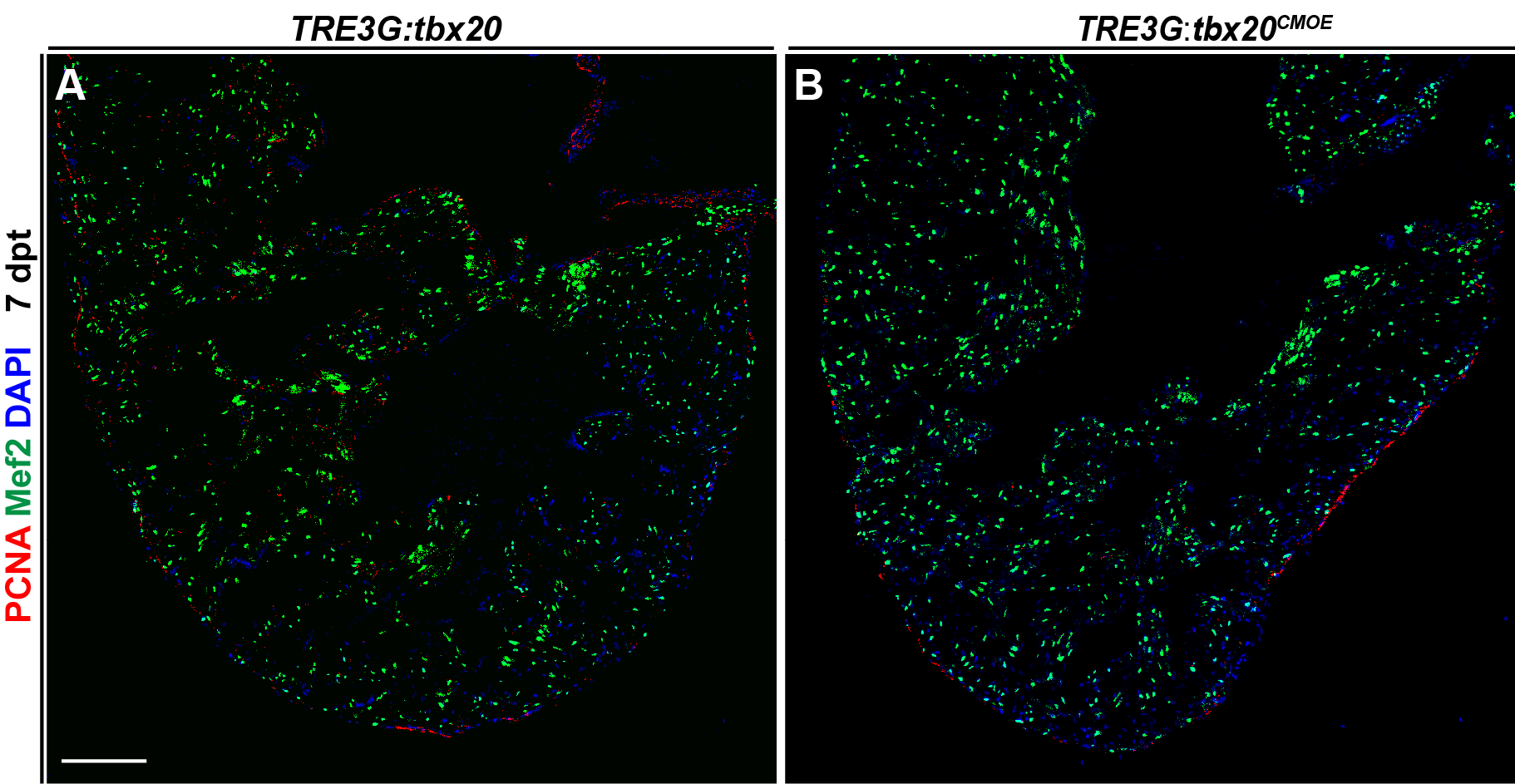

Supplement: FIGURE S4 — Proliferative CMs are almost not detectable in uninjured adult hearts with myocardial tbx20 overexpression. (A,B) Confocal fluorescence images of uninjured adult heart sections from Tg(TRE3G:tbx20) (A) and Tg(TRE3G:tbx20CMOE) zebrafish (B) after 7 days of DOX treatment co-stained with PCNA (red) and Mef2 (green) antibody. Scale bar: 100 μm. [file Image_4.TIF]

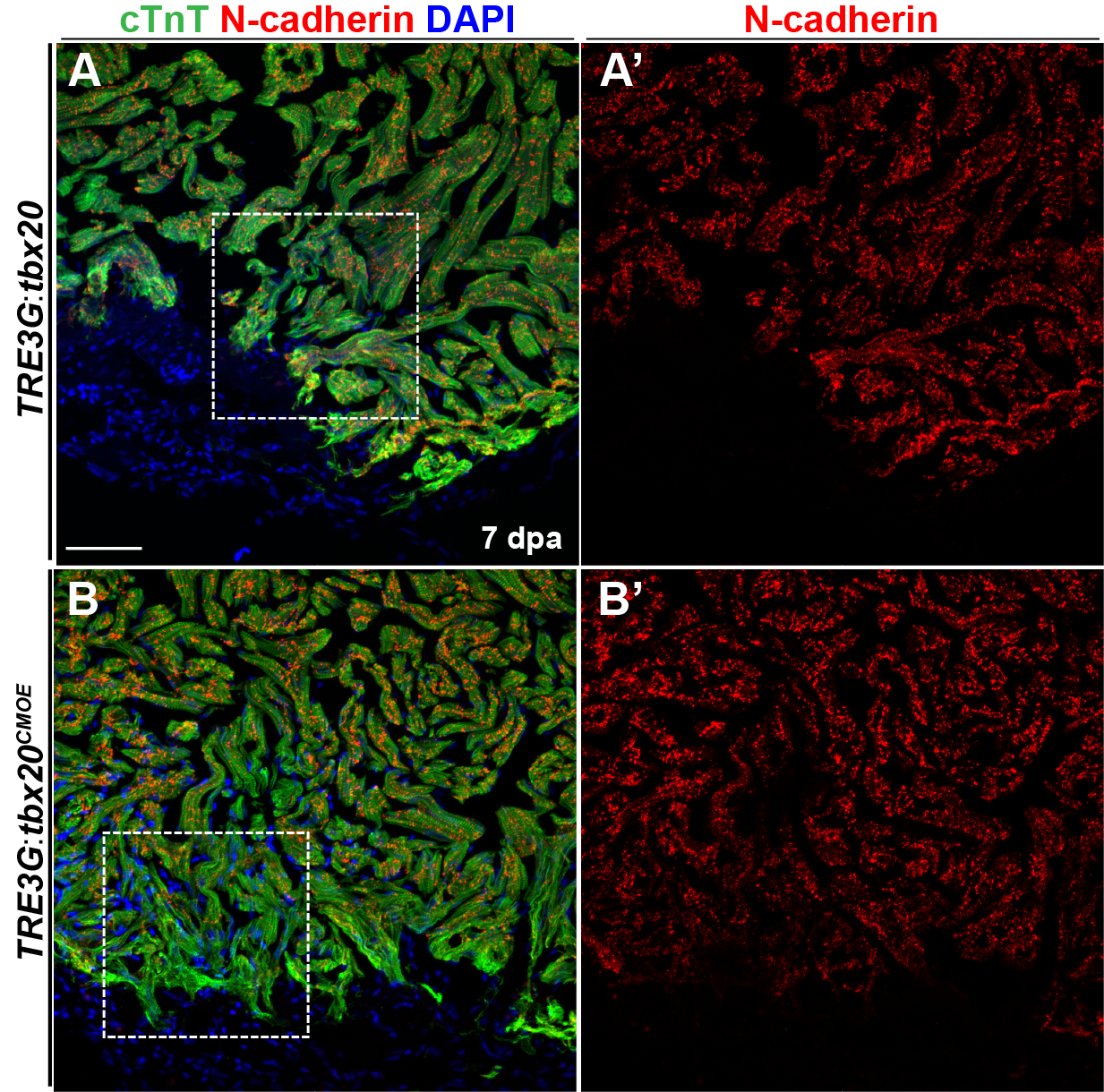

Supplement: FIGURE S5 — Myocardial tbx20 overexpression resulted in loss of cell-cell contact in the border zone myocardium. (A,B) Representative confocal fluorescence images of injured ventricle sections from Tg(TRE3G:tbx20) (A) and Tg(TRE3G:tbx20CMOE) (B) zebrafish co-stained with antibodies against cTnT (green) and N-cadherin (red). DAPI was used to stain nuclei. Boxed areas are locations of magnified images in Figures 3C,D. Scale bar: 100 μm. [file Image_5.TIF]

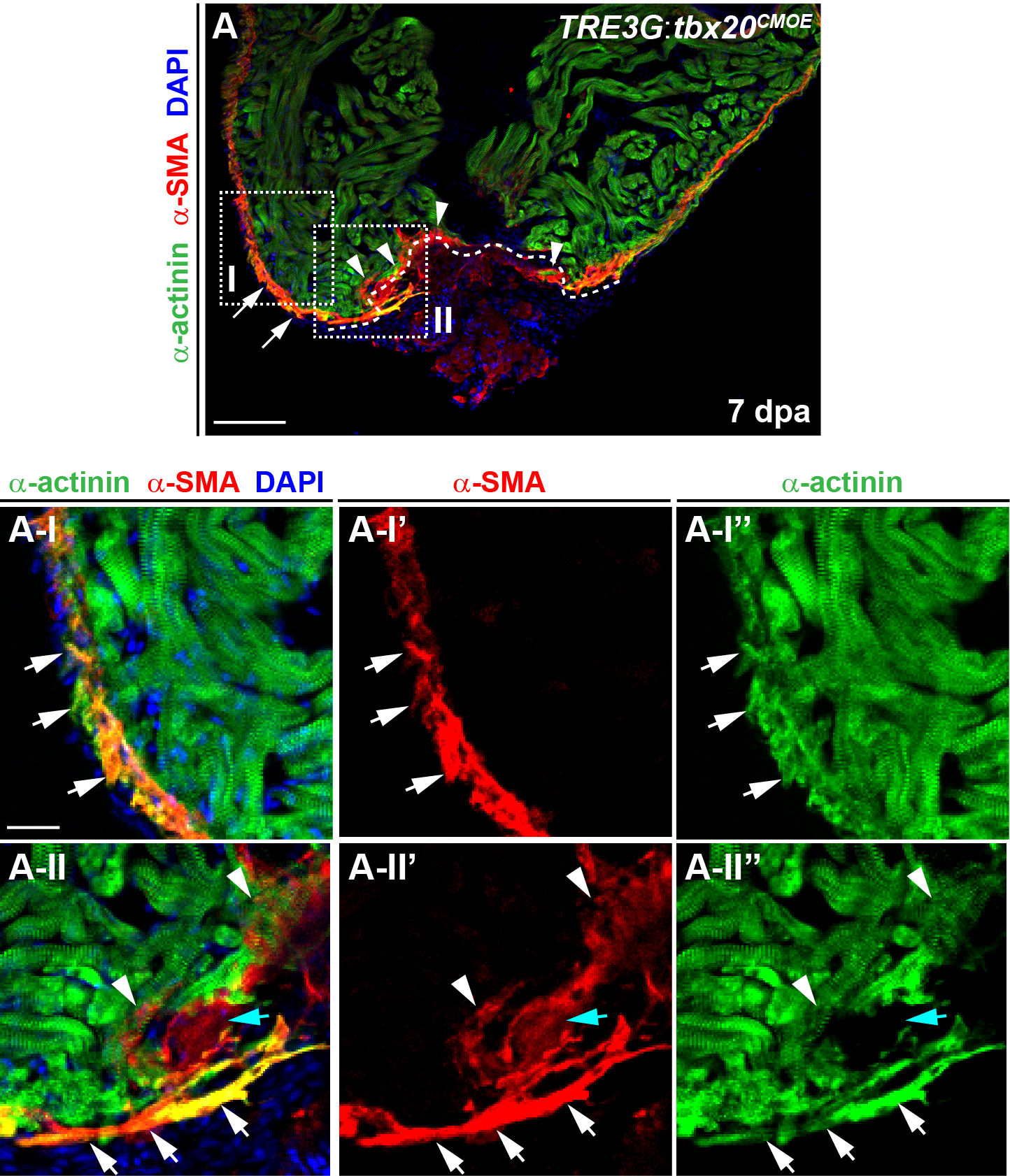

Supplement: FIGURE S6 — Myocardial tbx20 overexpression promotes injury-induced α-SMA in CMs at border zone and compact layer. (A) Representative confocal fluorescence image of injured ventricle sections from Tg(TRE3G:tbx20CMOE) zebrafish co-stained with antibodies against α-SMA (red) and α-actinin (green). DAPI was used to stain nuclei. Boxed areas are magnified below with split channels. White arrows point to compact layer myocardium, white arrowheads indicate α-SMA+ CMs in regenerating trabecular layer, light blue arrows point to non-cardiomyocyte cells. Scale bar: 100 μm (A); 20 μm (A-I–A-II”). [file Image_6.TIF]

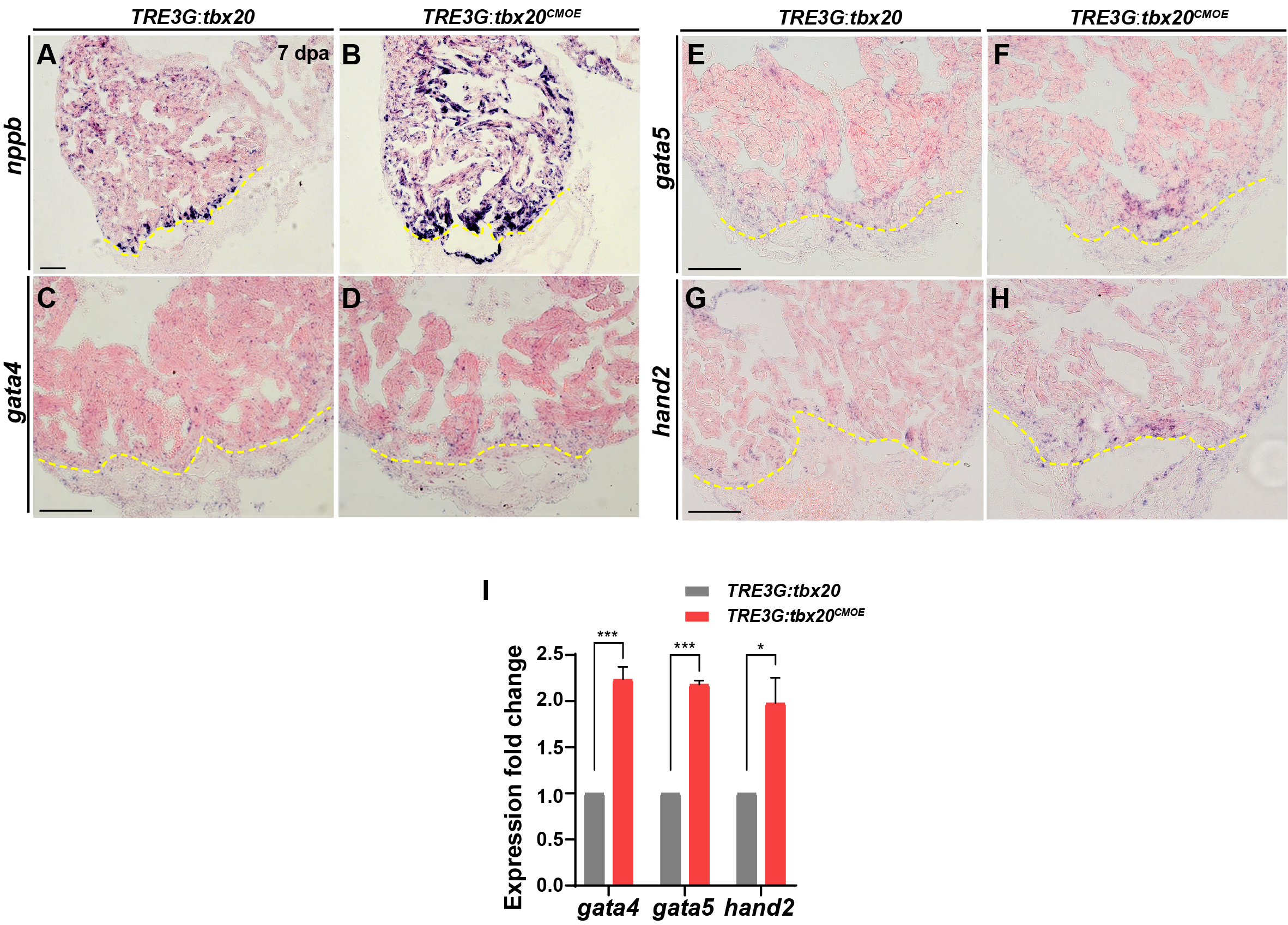

Supplement: FIGURE S7 — Myocardial tbx20 overexpression augments CMs dedifferentiation following injury. (A–H) Representative ISH images showing expression of nppb (A,B), gata4 (C,D), gata5 (E,F) and hand2 (G,H) in the adult injured Tg(TRE3G:tbx20) and Tg(TRE3G:tbx20CMOE) zebrafish ventricles after DOX-treatment at 7 dpa. Dotted lines demarcate amputation plane. Sale bar: 100 μm. (I) Statistical analyses of qPCR for gata4, gata5 and hand2 in the injured ventricle apices from Tg(TRE3G:tbx20) and Tg(TRE3G:tbx20CMOE) zebrafish at 7 dpa. Data represent three biological replicates. Mean ± SEM, ∗p < 0.05, ∗∗∗p < 0.001. [file Image_7.TIF]

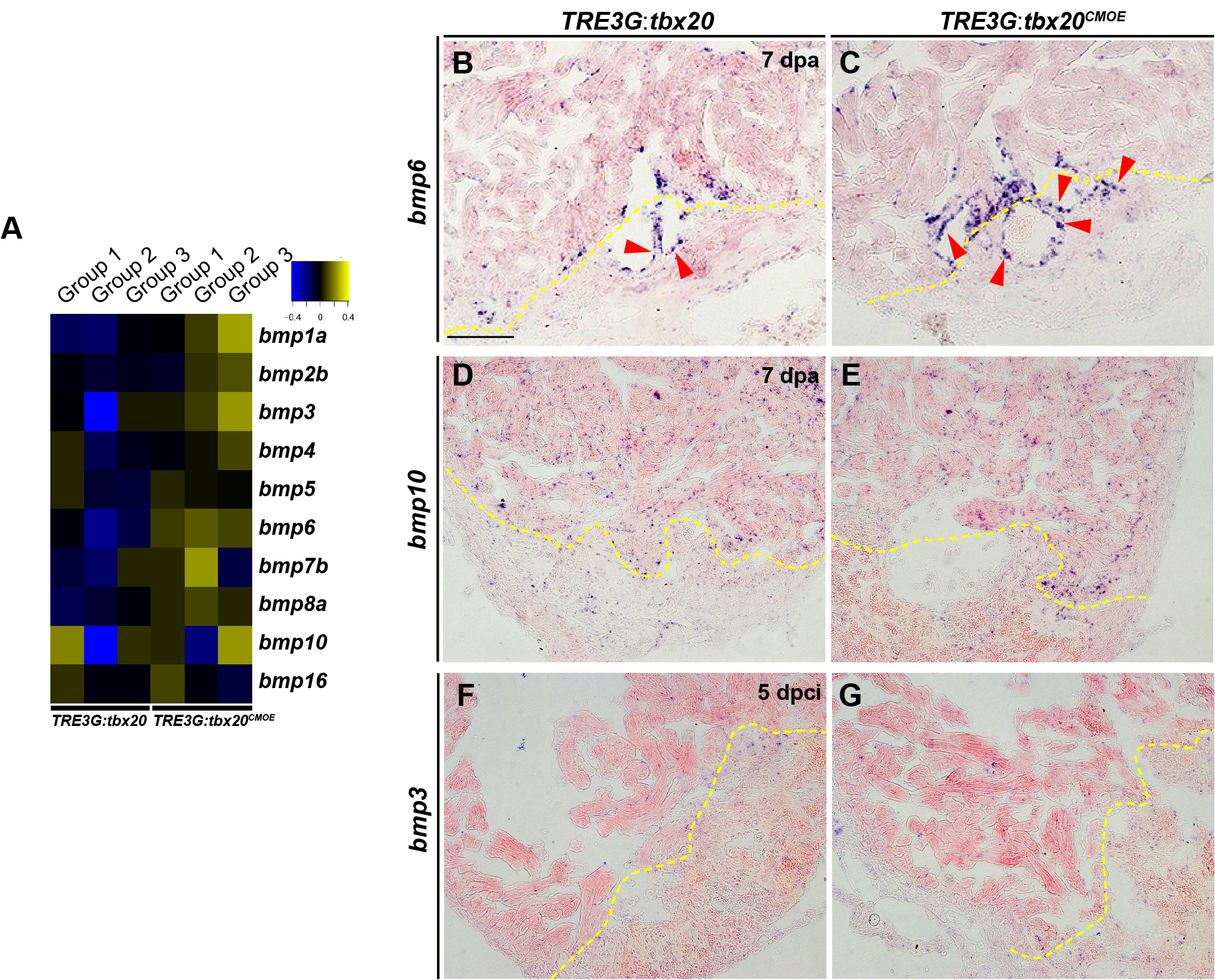

Supplement: FIGURE S8 — Expression profile of different BMP ligands in injured hearts. (A) Heat map showing fold changes for transcripts of BMP signaling ligands in DOX-treated Tg(TRE3G:tbx20) and Tg(TRE3G:tbx20CMOE) at 7 dpa detected by RNA-seq. (B–G) ISH analyses for bmp3 (F,G), bmp6 (B,C) and bmp10 (D,E) on heart sections from DOX-treated Tg(TRE3G:tbx20) or Tg(TRE3G:tbx20CMOE) zebrafish at 7 dpa and 5 dpci. Red arrowheads in (B,C) indicate endocardium with bmp6 signal. Dotted lines demarcate amputation plane. Sale bar: 100 μm. [file Image_8.TIF]

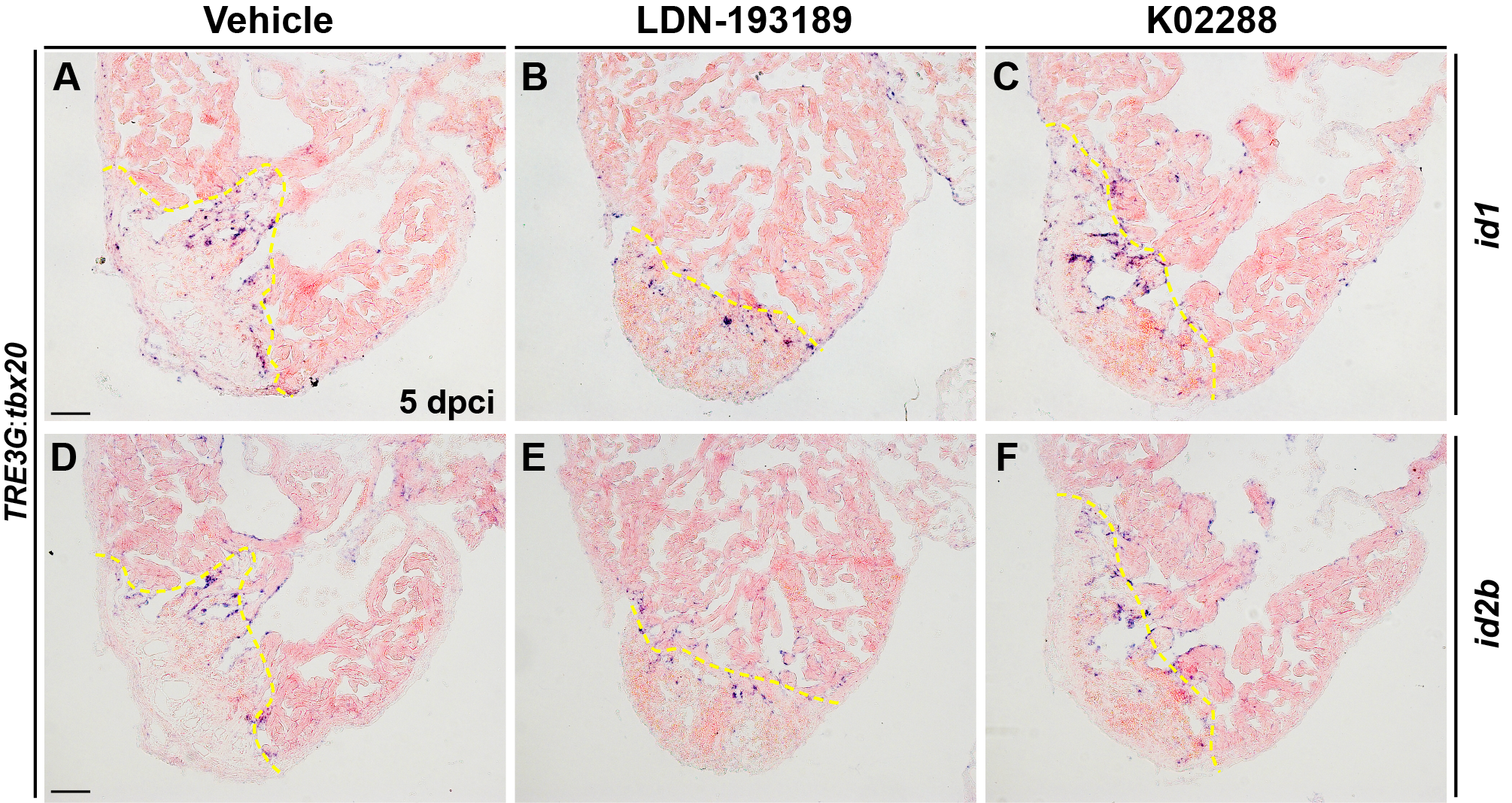

Supplement: FIGURE S9 — id1 and id2b expression are not altered in injured control hearts after BMP inhibitors treatment. (A–F) Representative ISH images showing expression pattern of id1 (A–C) and id2b (D–F) in DOX treated Tg(TRE3G:tbx20) fish at 5 dpci after vehicle or inhibitors treatment. Dotted lines demarcate amputation plane. Scale bar: 100 μm. [file Image_9.TIF]
